# Ethylene and hydrogen peroxide are involved in brassinosteroid-induced salt tolerance in tomato

**DOI:** 10.1038/srep35392

**Published:** 2016-10-14

**Authors:** Tong Zhu, Xingguang Deng, Xue Zhou, Lisha Zhu, Lijuan Zou, Pengxu Li, Dawei Zhang, Honghui Lin

**Affiliations:** 1 Ministry of Education, Key Laboratory for Bio-Resource and Eco-Environment, College of Life Science, State Key Laboratory of Hydraulics and Mountain River Engineering, Sichuan University, Chengdu, Sichuan, China

## Abstract

Crosstalk between phytohormone pathways is essential in plant growth, development and stress responses. Brassinosteroids (BRs) and ethylene are both pivotal plant growth regulators, and the interaction between these two phytohormones in the tomato response to salt stress is still unclear. Here, we explored the mechanism by which BRs affect ethylene biosynthesis and signaling in tomato seedlings under salt stress. The activity of 1-aminocyclopropane-1-carboxylate synthase (ACS), an ethylene synthesis enzyme, and the ethylene signaling pathway were activated in plants pretreated with BRs. Scavenging of ethylene production or silencing of ethylene signaling components inhibited BR-induced salt tolerance and blocked BR-induced activities of several antioxidant enzymes. Previous studies have reported that BRs can induce plant tolerance to a variety of environmental stimuli by triggering the generation of H_2_O_2_ as a signaling molecule. We also found that H_2_O_2_ might be involved in the crosstalk between BRs and ethylene in the tomato response to salt stress. Simultaneously, BR-induced ethylene production was partially blocked by pretreated with a reactive oxygen species scavenger or synthesis inhibitor. These results strongly demonstrated that ethylene and H_2_O_2_ play important roles in BR-dependent induction of plant salt stress tolerance. Furthermore, we also investigated the relationship between BR signaling and ethylene signaling pathways in plant processes responding to salt stress.

Environmental stresses such as temperature, drought, high salinity and other biotic or abiotic stresses influence plant growth and development. In response to various stresses, plants have evolved intricate defense mechanisms to increase their stress tolerance. Phytohormones, such as auxins, gibberellins, abscisic acid, cytokinins, salicylic acid, ethylene, jasmonates, brassinosteroids (BRs) and some peptide hormones are effective regulatory factors that are involved in plant adaptation to biotic and abiotic stresses by mediating a wide range of adaptive responses^1^,^2^,^3^,^4^,^5^,^6^. There are also other molecules and metabolically functional elements, such as reactive oxygen species (ROS)^7^ and alternative oxidase (AOX)^8^,^9^, that are involved in the adaption of plants to environmental stresses. Although these hormones and molecules play important roles in plant resistance against various environmental stimuli, the crosstalk of these signaling pathways and the relationship between these pivotal factors are still unclear.

BRs, a class of plant polyhydroxy steroids, participate in plant growth and developmental processes such as cell expansion and division, senescence and photomorphogenesis. Apart from their roles in the regulation of plant growth and development, BRs have been implicated in the regulation of various stress responses^10^,^11^. The upstream and downstream elements of the BR signaling pathway and their functions have been elucidated. Briefly, the plasma-membrane-localized and leucine-rich-repeat receptor kinase BR insensitive 1 (BRI1)^12^ perceives the BR signal, after which, via a series of phosphorylation and dephosphorylation events, the downstream transcription factors BRI1 EMS suppressor 1 (BES1) and brassinazole resistant 1 (BZR1) are activated resulting in the modulation of thousands of target genes^13^,^14^. The BR signaling pathway has also been connected with other signaling pathways, such as auxin, phytochrome and abscisic acid (ABA), in the response to environmental stimuli^15^. There is evidence to indicate that BRs also have some interaction with ethylene in various conditions, including environmental stresses, fruit ripening and seedling growth^15^,^16^,^17^,^18^.

Ethylene is another important plant hormone that plays a critical role in the regulation of stress responses. In *Arabidopsis*, ethylene is perceived by five receptors (ETR1, ETR2, ERS1, ERS2 and EIN4), and its downstream components such as CTR1, EIN2, EIN3 and ERF (ethylene responsive factor), among others, have also been identified for the ethylene signaling pathway^19^. In tomato, there are at least six ethylene receptors (ETR1-6)^20^. The predicted structures of the tomato receptor family are very similar to those in *Arabidopsis*. The downstream signaling elements in tomato have also been verified. There are at least three genes encoding proteins with significant homology to CTR1 in tomato. *SlCTR1* has been shown to functionally complement the *Arabidopsis ctr1* mutation^21^. SlEIN2 is encoded by a single gene, similar to *Arabidopsis*. In the case of EIN3, there is a family of four tomato genes. The tomato genes appear to be functionally redundant and each of the *EIL1-3* genes was shown to functionally complement the Arabidopsis *ein3* mutation. EIN3, and presumably the EIL proteins, are rapidly degraded by the ubiquitin/proteasome pathway in the absence of ethylene but accumulate to much higher levels in ethylene-treated plants. The F-box proteins that mediate this degradation are themselves positively transcriptionally regulated by ethylene. The ethylene response factors (ERFs) influence various developmental processes and are also important for adaptation to biotic or abiotic stresses such as pathogen attack, wounding, UV irradiation, extreme temperature and drought^22^,^23^,^24^.

There is evidence to indicate that BRs could interact with auxin and induce ethylene production in *Arabidopsis thaliana*^17^. Yi *et al*.^25^ reported that BRs and auxin differentially regulated the expression of the three members of the 1-aminocyclopropane-1-carboxylate synthase (ACS) family in mung bean (*Vigna radiata L*.). Studies have also shown that BRs could enhance the 1-aminocyclopropane-1-carboxylic acid synthase (ACS), thereby promoting ethylene accumulation^26^. Recent studies have shown that BR and its downstream signaling components such as Brassinazole resistant 1 (BZR1), are involved in fruit ripening via the regulation of ethylene accumulation^27^,^28^. Additionally, studies have provided evidence to support a connection between BRs and the ethylene signaling pathway. Moreover, whether BRs and ethylene are both involved in plant resistance to stress conditions and the relation between them are still unclear.

Tomato is one of the most important and widespread crops in the world. To date, few studies have focused on the relationship between BRs and the ethylene signaling pathway in the process of plant metabolism, especially in tomato plants under stress conditions. In our study, we found that BR treatment could enhance ethylene accumulation in tomato seedlings by promoting ACS activity, and this activity resulted in tomato seedlings that were resistant to salt stress. We also found that an H_2_O_2_ was involved in this process and H_2_O_2_ inhibitor or scavenger could partially block this process. Interestingly, virus induced gene silencing (VIGS) experiments showed that BR signaling molecules and ethylene signaling molecules might interact under salt stress. Furthermore, the possible relationship between BR and ethylene signaling in alleviating stress-induced damage was investigated.

## Results

### BRs enhance ethylene production in tomato seedlings

**Figure 1:**
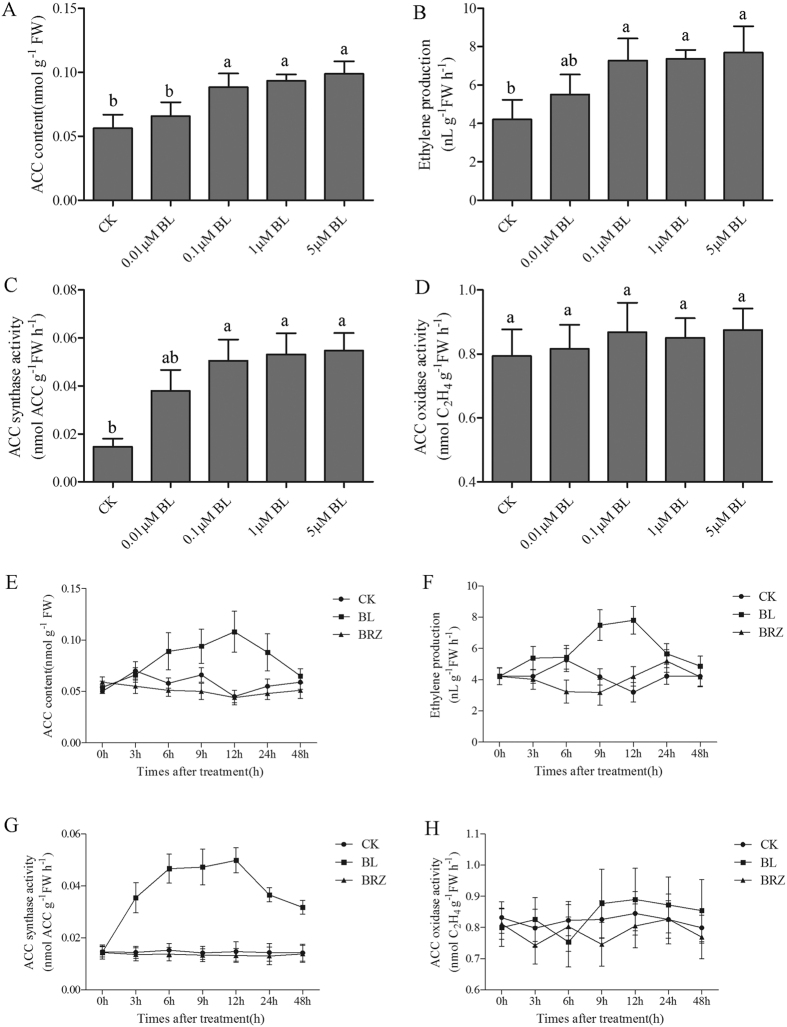
Effects of different BRs levels on ethylene accumulation. Tomato plants were sprayed with 0.01, 0.1, 1 or 5 μM BL solutions while the control plants were sprayed with distilled water or 1 μM BRZ. 12 h after treatment, the sixth leaf of tomato plants was used for ACC content (**A**), ethylene emission (**B**), ACS activity (**C**) and ACO activity (**D**) measurement. The time courses of BR-induced changes in ACC content (**E**), ethylene emission (**F**), ACS activity (**G**) and ACO activity (**H**). Tomato plants were sprayed with distilled water, 0.1 μM BL or 1 μM BRZ and the sixth leaf was harvested at the indicated time points for the assays. Bars represent the mean and standard deviation of values obtained from three biological repeats. Significant differences (P < 0.05) are denoted by different lowercase letters. FW, fresh weight.

To investigate whether BRs could influence ethylene production, we detected changes in ethylene emission, ACC content, ACS activity and ACO activity by using different concentrations of Brassinolide (BL), or brassinazole (BRZ) treatment for 12 h. When we sprayed tomato leaves with 0.01 μM, 0.1 μM, 1 μM or 5 μM BL solutions, the plants showed increased ACC content, ethylene emission and ACS activity; however, there was no increase in ACO activity (Fig. 1A–D). Although ACC content, ACS activity and ethylene emission all increased after treatment with different concentrations of BL, the increase in ACS activity was the most dramatic compared with others (Fig. 1C). Concomitantly, ethylene accumulation was significantly induced in the presence of BL. Concentrations of BL between 0.01 and 0.1 μM promoted obvious ethylene accumulation, but a further increase in BL concentration from 0.1 to 5 μM had no significant effects on additional increases in ethylene accumulation. This result indicated that a relatively high concentration of BL solution, (e.g., 5 μM) could not induce significantly more ethylene accumulation than a 0.1 μM BL solution. Based on these results, we used 0.1 μM BL in our subsequent experiments. To further confirm that BL could induce ethylene accumulation, we examined a time course of ethylene accumulation after BL treatment (Fig. 1E–H). As the results showed, ACC content (Fig. 1E), ethylene emission (Fig. 1F) and ACS activity (Fig. 1G) were significantly induced after 0.1 μM BL treatment with time, and the effect peaked at 12 h. By contrast, ACO activity only showed a slight increase at 9 h after BL treatment (Fig. 1H). In comparison, BRZ treatment had little effects on ethylene accumulation. Interestingly, the increase of ACS activity was earlier and more obvious than the increase of ACC and ethylene emission, which were induced by BRs. These results suggested that BR-induced ethylene accumulation was probably attributed to the BR-induced ACS activity. ACS is the key enzyme in ethylene synthesis, catalyzing the conversion of S-adenosyl-L-methionine (SAM) to 1-aminocyclopropane-1-carboxylate (ACC). ACC is then converted to ethylene by ACO. The transcriptional changes of genes in the ethylene pathway after BL treatment are shown in Fig. S1.

### BRI1 and its down-stream signal is involved in BR-induced ethylene accumulation

**Figure 2:**
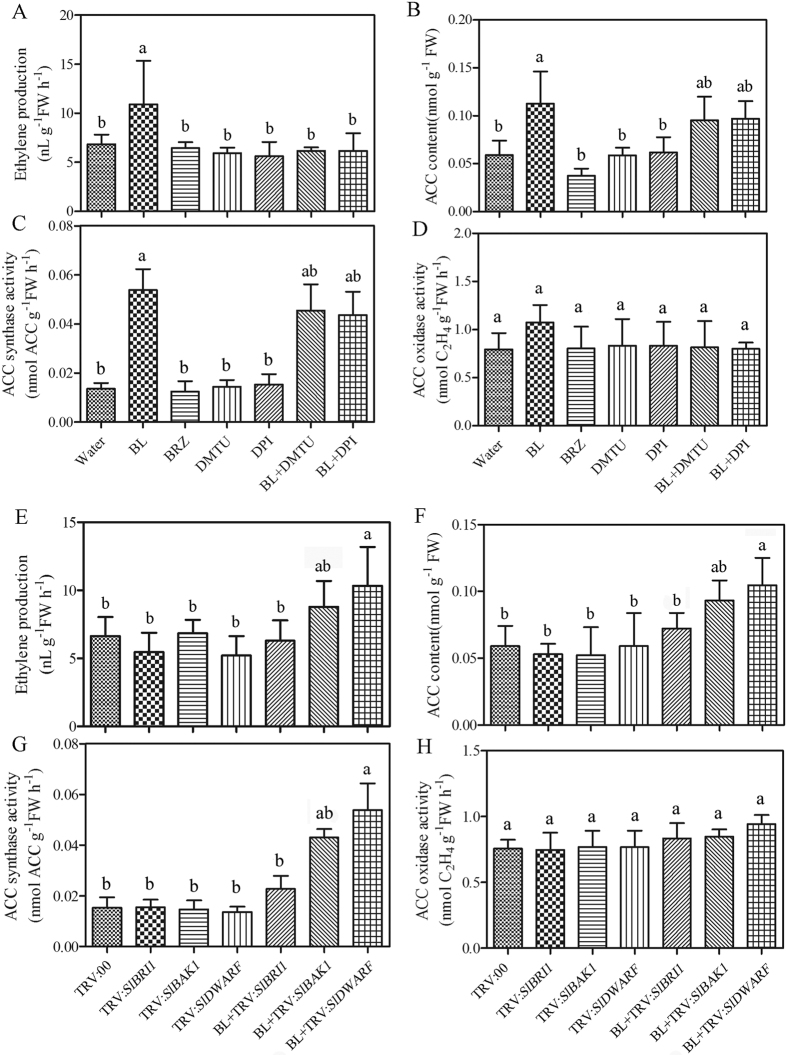
Changes of ethylene emission (**A**), ACC content (**B**), ACS activity (**C**) and ACO activity (**D**) in H_2_O_2_ scavenger DMTU or inhibitor DPI pre-treated plants as influenced by 0.1 μM BL. Tomato plants were treated with 5 mM DMTU for 8 h and then treated with 0.1 μM BL for another 12 h. Single treatment of BL or DMTU/DPI was included as control.Changes in ethylene emission (**E**) ACC content (**F**), ACS activity (**G**) and ACO activity (**H**) in *SlBRI1*-, *SlBAK1*- and *SlDWARF*-silenced plants as influenced by 0.1 μM BL. Involvement of H_2_O_2_ in the BR-induced ethylene accumulation. Bars represent mean and standard deviation of values obtained from three biological repeats. Significant differences (P < 0.05) are denoted by different lowercase letters. FW, fresh weight.

Next, we determined whether the BR signaling is involved in BR-induced ethylene accumulation. We used TRV-based VIGS system in tomato plants to silence BR insensitive 1 (BRI1), BRI1-Associated receptor kinase1/Somatic embryogenesis receptor kinase 3 (BAK1/SERK3), and 6-deoxocastasterone oxidase (DWARF). Silencing efficiency was confirmed by qRT-PCR 2 weeks after sprout vacuum-infiltration (Fig. S2). Database searches showed that tomato possessed the potential to encode at least the up-stream components of the canonical BR signal transduction pathway (Figs S3A,B, S4 and S5). As shown in Fig. 2, ACC content, ethylene emission, ACS activity and ACO activity were measured in silenced plants. These results demonstrated that in the *SlDWARF*-silenced plant, exogenous BL could enhance ethylene synthesis whereas in *SlBRI1*-scilenced plants, exogenous BL barely influenced ethylene accumulation. As BRI1 is an important BR receptor in tomato, when BRI1 was knocked down its down-stream signaling pathway would not be induced by BR treatment. These results suggest that BRI1 or its down-stream BR signaling molecules play an important role in BR-induced ethylene accumulation; that is, BRI1 or its downstream components may be involved in this process.

To confirm these results, we used bikinin, a chemical that effectively activates BRI1 downstream signaling, to trigger the BR signaling pathway instead of BL. In accordance with previous results, ethylene accumulation was significantly increased after bikinin treatment (Fig. S3C).

### The relationship between BR-induced H_2_O_2_ and ethylene accumulation

**Figure 3:**
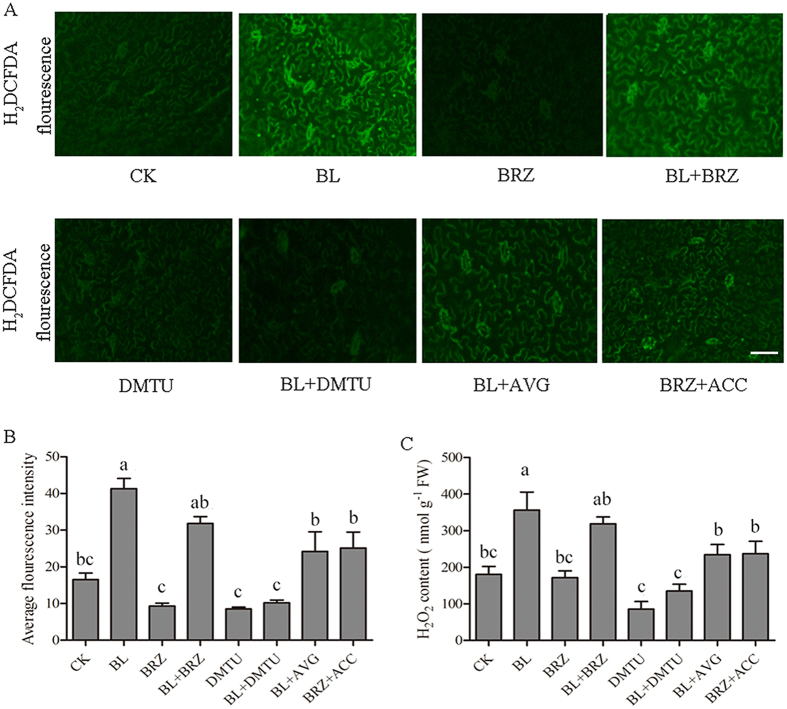
Responses of H_2_O_2_ generation in the sixth leaf of tomato plants by different chemical treatment. H_2_DCFDA staining and a fluorescence microscope were used for detection of H_2_O_2_ fluorescence. H_2_O_2_ increasing was showed by the green fluorescence at 12 h after treatments. Bars, 75 μm. Fluorescence imaging of H_2_DCFDA-loaded leaves (**A**). Average fluorescence intensity (**B**). H_2_O_2_ content (**C**). Bars represent the mean and standard deviation of values obtained from three biological repeats. Significant differences (P < 0.05) are denoted by different lowercase letters. FW, fresh weight.

As ROS play important roles in several phytohormone signaling pathways, we investigated the effects of BR-induced H_2_O_2_ on BR-induced ethylene accumulation. The H_2_O_2_ level was measured using 2′,7′-dichlorofluorescin diacetate (H_2_DCF-DA) as a fluorescence probe (Fig. 3A). The fluorescence intensity represents the internal H_2_O_2_ level. Compared with CK plants, the fluorescence level was much higher in BL-pretreated plants and, the fluorescence level was much lower in BRZ-pretreated plants. This result suggested that the internal H_2_O_2_ generation was triggered by exogenous BL and BRZ + BL treatment further verified this result. To determine the relationship between BR-induced H_2_O_2_ and ethylene accumulation, we first used 5 μM aminoethoxyvinylglycine (AVG) to inhibit the ethylene production and then treated the tomato seedlings with 0.5 μM BL. The results showed that the fluorescence level was increased but not as high as BL treatment, which means that the BR-induced H_2_O_2_ generation was impaired when ethylene synthesis was blocked. Interestingly, when we first used 1 μM BRZ to block BR production followed by 10 μM ACC to promote ethylene generation, we found that there was still some fluorescence (Fig. 3A,B). Therefore, these results demonstrated that ethylene could also trigger H_2_O_2_ generation. To further test whether H_2_O_2_ is involved in BR-induced ethylene accumulation, we used Dimethylthiourea (DMTU) to scavenge H_2_O_2_ or used DPI to inhibit H_2_O_2_ generation, and then we examined the ethylene synthesis capacity after BL treatment. As shown in Fig. 2, ACC content, ethylene emission and ACS activity in plant treated with BL + DMTU and BL + DPI (diphenylene iodonium, an NADPH oxidase inhibitor) were slightly higher than in water- treated plants (Fig. 2A,C). However, these increasing levels were much lower compared with BL treated plants, which demonstrated that BR-induced ethylene accumulation was blocked, at least to a certain extent. However, ACO activity was only slightly altered after such treatments (Fig. 2D). These results illustrate that BR-induced H_2_O_2_ generation is a part of BR-induced ethylene accumulation and that ethylene could also promote H_2_O_2_ generation. To confirm the results, we determined the H_2_O_2_ content in plants subjected to different treatments and the results are consistent with the above results (Fig. 3C).

### BRs enhanced tomato seedling tolerance to salt stress condition

**Figure 4:**
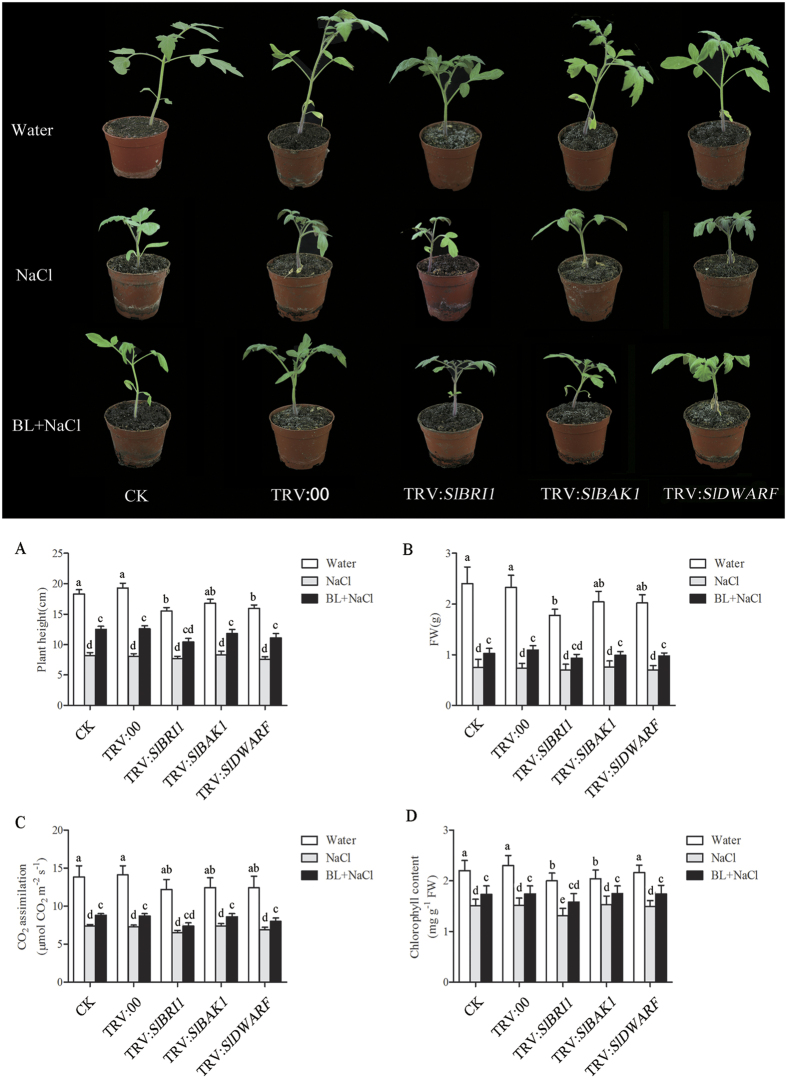
Phenotypes of *SlBRI1*-, *SlBAK1* and *SlDWARF*-silenced and corresponding empty vector control plants treated with water or 200 mM NaCl for 21 d. The seedlings were treated with water or 0.1 μM BL from the cotyledon stage to the six-leaf stage under salt condition. Plant height (**A**), fresh weight (FW) (**B**), CO_2_ assimilation rate (**C**) and chlorophyll content (**D**) of the silenced plants determined after 21 d of water or NaCl treatment with or without 0.1 μM BL pretreatment. Bars represent the mean and standard deviation of values obtained from three biological repeats. Significant differences (P < 0.05) are denoted by different lowercase letters. FW, fresh weight.

To determine the roles of BR and its signaling molecules in stress tolerance, *SlBRI1-*, *SlBAK1-* and *SlDWARF-*silenced tomato plants were challenged with salt stress. The salt tolerance of the silenced plants was determined by measurement of physiological parameters 3 weeks after 200 mM NaCl treatment. The phenotype of silenced tomato seedlings are shown in Fig. 4. In addition, the plants grown under salt stress and pretreated with BL presented better growth phenotype than plants pretreated with water alone. When treated with 200 mM NaCl, the tomato seedlings exhibited reduced growth, biomass, chlorophyll content and photosynthetic rate, whereas BL- pretreaed plants showed a better performance regarding these indices (Fig. 4A–D). Therefore, BRs could obviously enhance the tolerance of tomato seedlings to high salinity. However, in BL- pretreated TRV:*SlBRI1* plants, the physiological parameters were lower compared with other silenced plants or control plants under salt stress, which indicated that BR-induced salt tolerance was blocked in TRV:*SlBRI1* plants (Fig. 4). These results demonstrated that exogenous BL could enhance tomato plants salt tolerance and BR signaling molecules play an important role in BR-induced salt stress tolerance.

### Ethylene signaling is involved in BR-induced salt stress tolerance

**Figure 5:**
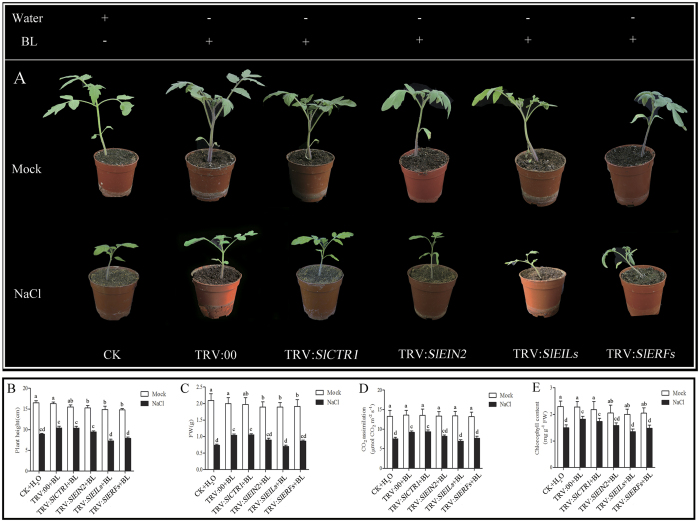
Phenotypes of *SlCTR1*-, *SlEIN2-*, *SlEILs-* and *SlERFs*-silenced and corresponding empty vector control plants treated with water or 200 mM NaCl for 21 d influenced by 0.1 μM BL (**A**). Plant height (**B**), fresh weight (FW) (**C**), CO_2_ assimilation rate (**D**) and chlorophyll content (**E**) of the silenced plants determined after 21 d of water or NaCl treatment. Bars represent the mean and standard deviation of values obtained from three biological repeats. Significant differences (P < 0.05) are denoted by different lowercase letters. FW, fresh weight.

To study the relationship between BR and ethylene signaling in BR-induced salt stress tolerance, we first used 1-methylcyclopropene (1-MCP) pretreatment to monitor the BR-induced salt tolerance. The results showed that when ethylene signaling pathway is blocked, the BR-induced salt tolerance was reversed (Fig. S6). Then, we constructed *SlCTR1*, *SlEIN2*, *SlEILs*, *SlERFs*-silenced plants using VIGS. To co-silence all of the members of the *SlEIL* gene family to prevent functional redundancy of these genes, we constructed a co-silencing vector that targeted three *SlEIL* genes, *SlEIL-1/2/3*, based on a 478 bp fragment of the *SlEIL3* ORF. qRT-PCR results revealed that the transcripts of the three EIL genes were down-regulated significantly in the TRV2:*SlEILs*-inoculated plants compared with the control plants infected with TRV empty vector (Fig. S7). As Fig. 5 shows, silencing of ethylene signaling molecules impaired BR-induced salt stress tolerance. The tomato seedlings growth, biomass, chlorophyll content and photosynthetic rate were lower in BL + TRV:*SlEIN2*, BL + TRV:*SlEINLs* and BL + TRV:*SlERFs* plants compared with BL + TRV:00 and BL + TRV:*SlCTR1* plants under salt conditions. In addition, in BL + TRV:*SlEILs* plants, the tomato seedlings exhibited severe growth defects (Fig. 5B–E).

**Figure 6:**
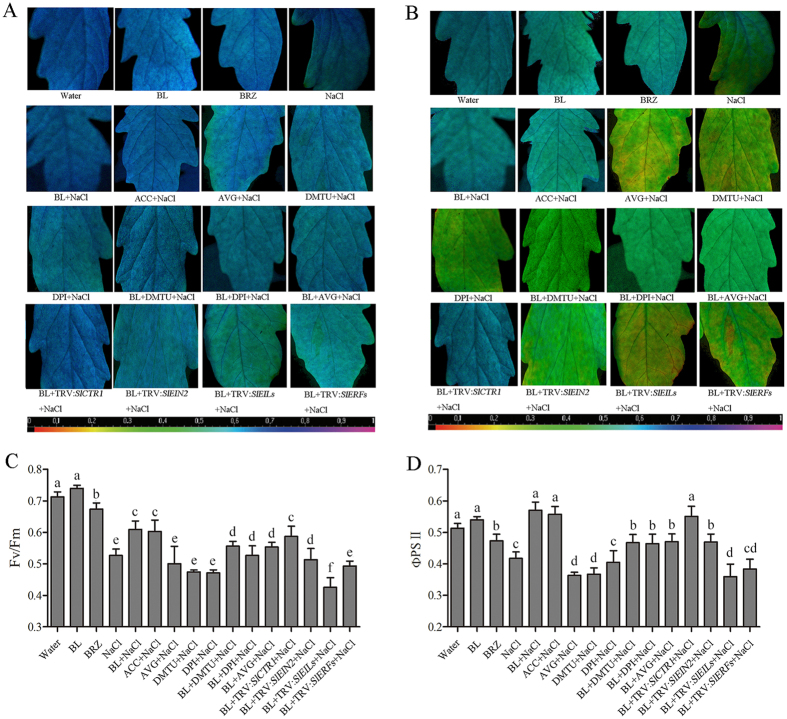
Photosystem damage in BR-induced salt stress tolerance. Images of the photochemical efficiency (F_v_/F_m_) (**A**) and maximum quantum yield of photosystem II (PS II) (**B**) in the sixth leaf of each tomato plant under salt stress for 5 d. Everage values of Fv/Fm (**C**) and ΦPS II (**D**) for the respective chlorophyll fluorescence images. The chemical inhibitor pretreated or silenced plants were first treated with 0.1 μM BL and then exposed to 200 mM NaCl for 3 d. At least nine plants were used for each treatment and a picture of one representative leaf is shown. Bars represent mean and standard deviation of values obtained from the six independent plants. Significant differences (P < 0.05) are denoted by different lowercase letters.

To further investigate the roles of ethylene signaling molecules in BR-induced salt stress tolerance, we tested the photochemical efficiency (F_v_/F_m_) and maximum quantum yield of photosystem II (PSII), which reflects the tomato seedlings photosystem (Fig. 6). We first analyzed the effects of AVG (an ACS inhibitor) in BR-induced salt stress tolerance. The results showed that in BL-treated plants, the F_v_/F_m_ was higher than that of water-treated plants under salt stress conditions, which was consistent with ΦPS II (Fig. 6A,B). However, BR-induced salt tolerance, expressed as F_v_/F_m_, was obviously inhibited when plants were pretreated with AVG. To further investigate the role of ethylene signaling molecules in BR-induced salt stress tolerance, we examined the F_v_/F_m_ and ΦPS II in TRV:*SlCTR1*, TRV:*SlEIN2*, TRV:*SlEILs* and TRV:*SlERFs* plants pretreated with BL under salt stress. Similar to AVG pre-treatment, in BL-pretreated TRV:*SlEIN2*, TRV:*SlEILs* and TRV:*SlERFs* plants, the F_v_/F_m_ and ΦPS II were significantly lower than BL pretreated TRV:00 plants under salt stress, particularly in TRV:*SlEILs* plants (Fig. 6A,B). Interestingly, we also found that when H_2_O_2_ was scavenged or its synthesis were inhibited by DMTU or DPI pretreatment, the BR-induced salt tolerance were partially blocked as the F_v_/F_m_ in BL + DMTU + NaCl−or BL + DPI + NaCl−treated plants was lower than BL + NaCl−treated plants. These results showed that the ethylene signaling pathway and H_2_O_2_ were both involved in BR-induced salt stress tolerance.

Furthermore, better understanding the cross-talk between BL and ethylene under salinity stress, we used BR silenced plants treated with ACC to obtain a complete picture of BL and ethylene interplay. As shown in Fig. S8, after ACC treatment, the BR silenced plants still exhibited better growth than untreated plants, indicating that ethylene works down-stream in BR-induced salt tolerance. In other words, these results further confirmed that ethylene participates in BR-induced salt stress tolerance.

### The ethylene signaling pathway is involved in BR-induced alleviation of oxidative damage and increases antioxidant enzyme capacity

**Figure 7:**
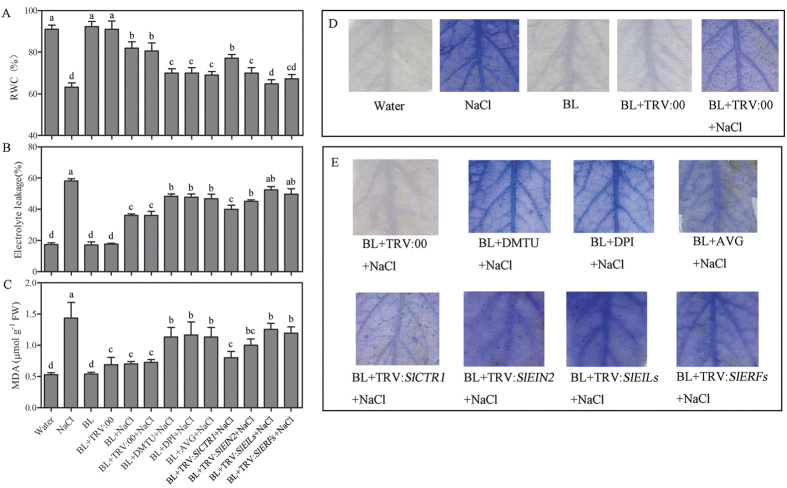
Ethylene signal pathway is involved in BR-induced alleviates of oxidative damage. Quantitative measurements of RWC (**A**). Quantitative measurements of EL (**B**). Quantitative measurements of MDA content (**C**). Trypan blue (1.25 mg ml^−1^) staining was used to detect the degree of cell death (**D**). The chemical inhibitor pretreated or silenced plants were first treated with 0.1 μM BL and then exposed to 200 mM NaCl for 5 d. Experiments were repeated at least three times with similar results. Bars represent mean and standard deviation of values obtained from three biological repeats. Significant differences (P < 0.05) are denoted by different lowercase letters. FW, fresh weight.

Previous studies have suggested that BR could alleviate oxidative damage in plants in response to various abiotic stresses. Here, we monitored the relative water content (RWC), electrolyte leakage (EL), malondialdehyde (MDA) content and the level of cell death of tomato seedlings under NaCl treatment (Fig. 7). Similar to previous results, the RWC was lower in BL pretreated TRV:*SlEIN2*, TRV:*SlEILs* and TRV:*SlERFs* plants compared with the other plants under salt stress (Fig. 7A). The EL and MDA content significantly increased in these plants compared with the other plants (Fig. 7B,C). We also detected the Typan Blue staining to monitor the level of cell death. As Fig. 7D shows, the staining was obviously deeper in BL-pretreated TRV:*SlEIN2*, TRV:*SlEILs* and TRV:*SlERFs* plants under salt condition. These results demonstrated that the BR-induced alleviation of oxidative damage was partly diminished when the ethylene signaling pathway was blocked.

**Figure 8:**
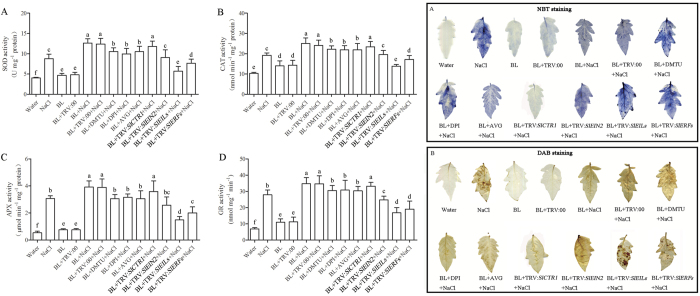
Changes in the activities of SOD (**A**), CAT (**B**), APX (**C**), GR (**D**) in tomato plants under salt stress. The chemical inhibitor pretreated or silenced plants were first treated with 0.1 μM BL and then exposed to 200 mM NaCl for 5 d. Superoxide contents were detected by 0.5 mg/mL nitro blue tetrazolium (NBT) staining (**E**). H_2_O_2_ levels were detected by 2 mg/mL 3,3-diaminobenzidine (DAB) staining (**F**). Ten plants were used for each treatment and a picture of one representative leaf is shown. Bars represent mean and standard deviation of values obtained from three biological repeats. Significant differences (P < 0.05) are denoted by different lowercase letters.

ROS generation is a common response when plants are exposed to environmental stresses. We detected the accumulation of superoxide and H_2_O_2_ using NBT and DAB staining, respectively (Fig. 8E,F). BL pretreatment decreased the superoxide and H_2_O_2_ generation under salt stress but increased when pretreated with AVG, DMTU and DPI or when the ethylene signaling pathway was inactivated by the VIGS approach.

We next monitored the activity of antioxidant enzymes, which plays key roles in plants to antagonize oxidative damage. As Fig. 8A–D shows, the activity of SOD, CAT, APX and GPX were significantly increased under salt stress and the increase was elevated by BL pretreatment. Moreover, the BR-induced increase of antioxidant enzyme activities was blocked when ethylene production, H_2_O_2_ generation or the ethylene signaling pathway was inhibited by chemical inhibitors or gene silencing. Therefore, exogenous BRs induced plant responses to salt stress in an H_2_O_2_ and ethylene-dependent manner.

## Discussion

Several studies have shown that BRs play a pivotal role in plant response to various stresses. In our study, we provided evidence that H_2_O_2_, ethylene and its signaling molecules are involved in BR-induced salt stress tolerance. In BL-treated tomato seedlings, we observed an increase in H_2_O_2_ content and ethylene production. Our results demonstrated that BR-induced H_2_O_2_ generation was a part of BR-induced ethylene accumulation and ethylene could also in turn promote H_2_O_2_ generation. The H_2_O_2_ and ethylene accumulation, which was promoted by exogenous BL treatment, appeared to be positive signals that contributed to the alleviation of oxidative damage in tomato. In addition, ethylene signaling molecules were essential components of BR-induced salt tolerance in tomato. Tomato seedlings pretreated with BL demonstrated better growth under salt stress than plants that were pretreated with water alone. Furthermore, the antioxidant system was activated by exogenous BL treatment. Taken together, these studies support the involvement of BRs in plant responses to salt stress.

It is well known that, crosstalk between plant hormones participates in the regulation of various processes of plant growth and development. For instance, BR and auxin signaling transcription factors participate in the promotion of hypocotyl cell elongation^29^. “Triple response”, is a well- documented ethylene response of etiolated seedlings, i.e., short, thickened hypocotyl and root and exaggerated curvature of the apical hook^30^. Therefore, auxin, BRs and ethylene are functioning in the early development of seedlings. There is also genetic and molecular evidence for the crosstalk between BRs and ABA in tomato stress tolerance^31^. Studies have indicated that interactions must exist between BR and ethylene in many aspects of plants development^32^. Hansen *et al*. reported that BRs increased the stability of two type 2 ACS proteins^26^. Studies of mung bean demonstrated that the transcription level of *VrACS7* was regulated by BRs^25^. Recent research reports that ethylene is an important phytohormone in the response to several abiotic stresses and revealed the action of its signaling pathway in plant abiotic stress tolerance^33^. Xia *et al*. reported that upon perception of BR signal, NADPH oxidases might be activated to generate ROS, which initiates a protein phosphorylation cascade^34^. There are also studies reporting that H_2_O_2_ is critical for brassinosteroid (BR)- and abscisic acid (ABA)-induced stress tolerance in tomato^35^. Nie *et al*. reported that silencing of tomato RBOH1 and MPK2 abolishes brassinosteroid-induced H_2_O_2_ generation and stress tolerance^36^. Therefore, our results support the finding of previous studies. Studies have also shown that BL interaction with IAA could produce higher levels of ethylene than either alone^24^. Li *et al*. reported that overexpression of *DWARF* (*DWF*), the key BR biosynthetic gene, led to elevated ethylene production in tomato plants^18^. However, few studies have focused on the changes in ethylene biosynthesis and signaling genes. Therefore, there is still a need for more in-depth studies to explore how ethylene production is triggered by BRs. Although H_2_O_2_ and ethylene are both induced by BRs, the relation between them is still unclear. In the present study, we observed that both H_2_O_2_ and ethylene accumulation were increased after BL treatment in tomato. Tomato seedlings treated with different concentrations of BL solution exhibited a significant increase in ethylene accumulation (Fig. 1). We further monitored the time course of 0.1 μM BL treatment and confirmed the BR-induced ethylene accumulation mainly came from increased ACS capacity. The results showed that BR signaling molecules have participated in these processes. This finding is supported by results that show that BR-induced ethylene production almost vanished in *SlBRI1*-silenced plants, but not in *SlDWARF-*silenced plants (Fig. 2). BR signaling starts at the cell surface and is perceived by a small family of plasma membrane-localized leucine-rich repeat receptor kinases (LRR-RKs). BRASSINOSTEROID INSENSITIVE 1 (BRI1) is the founding member of this small family of receptors and accounts for most of the binding activities of BRs in Arabidopsis^37^,^38^. Therefore, it has been suggested that BRI1 or its down-stream molecules must be involved in the process of BR-mediated ethylene production. Interestingly, BL-pretreated *SlBAK1*-silenced plants showed a slight increase in ethylene accumulation. This effect is mainly due to the functional redundancy of other family members^39^,^40^. To investigate the relationship between H_2_O_2_ and ethylene upon exogenous BL treatment, we first used AVG to inhibit ethylene synthesis and then we monitored H_2_O_2_ generation that is induced by BRs. The results suggested that BR-induced H_2_O_2_ generation was partially blocked when ethylene synthesis was inhibited. BRZ + ACC treatment showed increased fluorescence, which suggested that ethylene could also trigger H_2_O_2_ generation. To detect whether BR-induced H_2_O_2_ generation is involved in BR-induced ethylene production, we used DMTU and DPI to scavenge or inhibit H_2_O_2_ generation. The ethylene accumulation induced by BRs was influenced when the plants were pretreated with these chemical inhibitors (Fig. 2A–D). These results demonstrated that BR could either promote ethylene production or H_2_O_2_ generation. In addition, our research suggested that BR-induced H_2_O_2_ was a part of BR-mediated ethylene production and that the ethylene production that is triggered by BL also participated in H_2_O_2_ generation. Evidence indicates that ethylene-induced stomatal closure is mainly due to the promoting of NADPH oxidase-mediated ROS production in stomatal guard cells^41^. There are also studies suggesting that H_2_O_2_ may be involved in the induction of ethylene biosynthesis genes^42^. Therefore, based on our results, we hypothesize that BR-induced ROS generation probably activates mitogen-activated protein kinases (MAPKs), which could enhance ACS stabilization and thus may enhance ACC generation and, in turn, affect ethylene production.

Currently, BRs have attracted increasing attentionfrom phytologists and are believed to play a critical role in stress alleviation. Several studies have revealed that BRs play an important role in enhancing plant tolerance to a variety of environmental stresses^43^,^44^. In our study, we provided several lines of evidence that BRs could also improve the tolerance of tomato response to salt stress. Furthermore, ROS and ethylene are very likely to participate in BR-induced salt stress tolerance.

ROS generation is regarded as a common cellular response during stressful conditions. H_2_O_2_ is considered a central signaling molecule in plant responses to various environmental stresses^45^. Form another point of view, ROS overproduction might causes serious oxidation damage which could influence the ordinary operation of plant metabolism^46^. Similar to other studies, we found that H_2_O_2_ also participates in BR-induced salt stress tolerance. When tomato plants were pretreated with DMTU or DPI, BL-induced salt tolerance was partially impaired (Figs 6, 7 and 8). It is interesting that there is a discrepancy in H_2_O_2_ generation, as shown in Figs 3 and 8. The BL- treated plants in Fig. 3 exhibited elevated H_2_O_2_ generation, whereas the BL- pretreated plants in Fig. 8 showed divergent results compared with the control. However, the time at which H_2_O_2_ was detected is different between Figs 3 and 8. BR-induced H_2_O_2_ generation reached peak levels at approximately 12–24 h after BL treatment, then decreased and was maintained at normal conditions, which we also noted in our previous study^9^. ROS generation is a common cellular response during stressful conditions. On the one hand, the generation of ROS is necessary for signaling in individual stress responses. On the other hand, ROS overproduction causes oxidation damage to cellular components, which is a common destructive effect of abiotic stress. Time courses experiments of the effects of BL on H_2_O_2_ accumulation showed that BRs induced ROS production mildly under normal growth conditions. Interestingly, when exposed to stress conditions, water-treated plants suffered a dramatic increase in H_2_O_2_ accumulation, forming a sharp contrast with BL-treated plants, which had a comparatively slight increase. This result is mainly because BL-treated plants have a sharper increase in alternative respiration and other defense system when compared with water-treated plants. The enhancement of these defense systems by BL pre-treatment might play a key role in ROS avoidance under stress conditions^9^.

Seldom has research focused on the relationship between BRs and ethylene under stress condition. Divi *et al*. reported that BR-mediated stress tolerance in *Arabidopsis* showed interactions with ethylene, salicylic acid and abscisic acid pathways. Some studies also supported the crosstalk between BRs and ethylene under stress conditions^47^. However, it is still unclear whether and how ethylene and its signaling molecules are involved in BR-mediated salt stress tolerance in tomato plants. In our study, we used AVG, a specific ACS inhibitor, to block ethylene accumulation before BL treatment under salt stress. The results showed that BR-induced salt tolerance was inhibited when ethylene synthesis was blocked in tomato plants (Figs 6, 7 and 8). Furthermore, the VIGS assay suggested that ethylene signaling molecules, such as EIN2, EILs and ERFs, also participated in BR-mediated salt tolerance (Fig. 5). The oxidative damage was obviously higher in *SlEIN2*, *SlEILs* and *SlERFs*-silenced BL + NaCl tomato plants than control BL + NaCl treatment plants. Interestingly, our results showed that in *SlEILs*-silenced plants, the damage was significantly higher than in any other plants (Figs 7 and 8). In *Arabidopsis*, the ethylene signaling pathway is well understood. As we all know, unlike the repressor CTR1, EIN2 is a central component of the ethylene signaling transduction pathway, and its null mutant *ein2* is completely insensitive to ethylene^48^. EIN3/EIL1 is positively regulated by EIN2 and the activated EIN3/EIL1 results in the activation of transcription of *ERF1*, the ethylene transcription factor, and other downstream genes^49^. Recent, studies have suggested that ethylene stabilizes EIN3/EIL1 mainly by promoting the proteasomal degradation of EBF1/EBF2, and EIN2 is indispensable for mediating ethylene-induced EIN3/EIL1 accumulation and EBF1/2 degradation^50^. However, the salt-induced EIN3/EIL1 protein stabilization was promoted by EBF1/EBF2 proteasomal degradation probably in an EIN2 independent manner, in Arabidopsis. In *Solanum lycopersicum*, four cDNAs encoding EIN3-like proteins were cloned from tomato and designated as *LeEIL1-4* 51,52. Each of the *LeEIL1-3* genes complemented the transgenic Arabidopsis *ein3* mutant, indicating that they are involved in ethylene signaling. Antisense suppression of one *LeEIL* gene in the tomato plant did not reduce ethylene sensitivity, whereas the reduction of the three *LeEILs* resulted in the loss of ethylene responses. In our study, we constructed a co-silencing vector that targeted three *SlEIL* genes, *SlEIL-1/2/3*, based on a 478 bp fragment of the *SlEIL3* ORF. When the EILs were silenced in tomato plants, BR-induced salt tolerance was severely affected (Figs 7 and 8). These results indicated that EILs-dependent transduction pathway was critical in BR-induced salt tolerance in tomato plants.

GSK3-like kinase (BIN2) is a key master regulator that transduces BR signals to a large repertoire of physiological targets that act in various aspects of plant development, metabolism and other signaling pathways. Previous studies have reported that BIN2 phosphorylates YODA (YDA) and MKK4, two proteins that act upstream of MPK3/6 in stomata patterning^53^,^54^. Once BIN2-mediated phosphorylation of YDA and MKK4 was released by BRs, the activation of MKK4 leads to the activation of MPK3/6. MAPKs play important roles in ET signaling, the MPK3/MPK6 pathway, and positively regulating EIN3, which is also required for the activation of ACS genes involved in ET biosynthesis^55^,^56^. In our study, we found an increased activity of ACS and transcriptional changes in ACS, ACO and ethylene signaling pathway genes which probably contributed to the elevated ACC and ethylene production (Figs 1 and S1). Meanwhile, an EILs-dependent signaling pathway is highly correlated with in BR-induced salt tolerance. Because MAP kinase pathway (MKK, MKK9 and MPK3/6) positively regulates EIN3 activity, and these pathways (e.g., mkk9) loss-of-function mutants lead to increased salt sensitivity, we suppose that MAPK pathway very likely participates in BR-induced ethylene accumulation and EILs-dependent salt tolerance which could be triggered by exogenous BL treatment in tomato plants.

**Figure 9:**
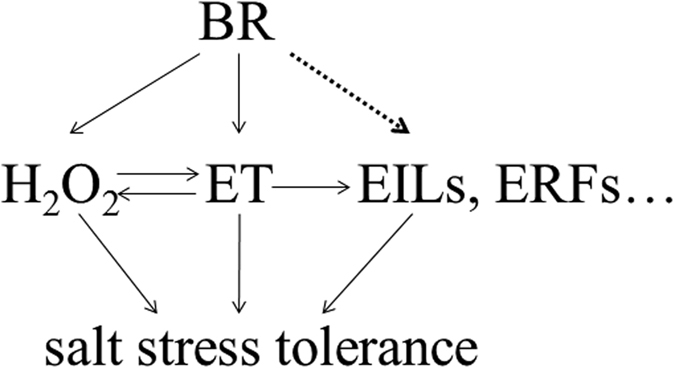
A proposed model for the induction of salt stress tolerance by BRs in tomato plants.

Taken together, we presented physiological and genetic evidence of the dynamic interplay between BRs and ethylene in tomato salt stress tolerance. Following the perception of BR signal, there appeared to be enhanced ethylene synthesis and EILs stabilization. At the same time, the increased ROS acting as a secondary messenger can also trigger increased ethylene biosynthesis and ROS down-stream responses. The elevated ethylene accumulation and activated EILs-dependent signaling pathway then activate specific defense responses (i.e., the AOX capacity, ERFs or the transcription of its responsive genes), which lead to salt stress tolerance (Fig. 9).

## Methods

### Plant material and growth conditions

Tomato (*Solanum lycopersicum* cv. Yuanbao) plants were grown in a growth room under the following conditions: 16/8 h day/night cycle, 25/20 °C day/night temperature (for VIGS plant, 23/18 °C day/night), 50 ± 10% humidity and 100 μmol m^−2^s^−1^ light intensity. After germination on water-soaked filter paper in Petri dish for 3 d in the dark, the sprouting seeds were transplanted in pots containing humus-vermiculite mixture (1:1, v/v), and watered every three days in subsequent culture until use. For salt stress tolerance measurement, plants were exposed to 200 mM NaCl for 3 d.

### Chemical treatments

Brassinolide (BL, the most active BR) and brassinazole (BRZ, a specific inhibitor of BR biosynthesis) were purchased from Wako Pure Chemical Industries (Chuo-Ku, Osaka, Japan) and Santa Cruz Biotechnology (Dallas, Texas, USA), respectively. 1-Aminocyclopropane-1-carboxylic acid (ACC; precursor of the ethylene biosynthesis) and ethylene biosynthesis blocker aminoethoxyvinylglycine (AVG) were purchased from Sigma-Aldrich (Shanghai, China). Dimethylthiourea (DMTU, an H_2_O_2_ scavenger), 1-methylcyclopropene (1-MCP) and diphenylene iodonium (DPI, an NADPH oxidase inhibitor) were purchased from Sigma (St Louis, USA). The hormone and inhibitor solutions were prepared in distilled water containing 0.02% (v/v) Tween 20. The chemicals and the concentrations used are as follows: BL, 0.01, 0.1, 1, and 5 μM; BRZ, 1 μM; DMTU, 5 mM; DPI, 100 μM; AVG, 5 μM. ACC, 10 μM. Distilled water containing 0.02% (v/v) Tween 20 was used as a control treatment. For DMUT/DPI + BL treatment, plants were first sprayed with 5 mM DMTU/100 μM DPI, and 8 h later they were sprayed with 0.1 μM BL for another 12 h. For BL + AVG or BRZ + ACC treatment, plants were first sprayed with 5 μM AVG or 10 μM ACC, and 8 h later they were sprayed with 0.1 μM BL or 5 μM BRZ for another 12 h. The plants were then exposed to 200 mM NaCl.

### VIGS vector construction

Virus-induced gene silencing (VIGS) of genes in tomato seedlings was conducted according to the previously described protocols using tobacco rattle virus with little modification^9^,^57^. The vector was kindly provided by Dr. GZ Qin (Key Laboratory of Plant Resources, Institute of Botany, Chinese Academy of Sciences,). To silence BR and ethylene signal elements, partial cDNA of *SlBRI1*, *SlBAK1*, *SlDWARF*, *SlCTR1*, *SlEIN2*, *SlEILs* and *SlERFs* were amplified by RT-PCR using primers (supplementary Table S1). The amplified fragments were then cloned into pTRV2 vector. For the VIGS assay, *Agrobacterium tumefaciens* strain GV3101 containing pTRV1, pTRV2 (with the inserted fragment) were used for RNAi. The infiltration mixture and germinating seeds (approximately 10 seeds) were placed in each 1.5 or 2.0 mL centrifuge tubes. Then, centrifuge tubes were placed into a vacuum dryer. Agrobacterium was infiltrated into sprouts using a sprout vacuum-infiltration system [a vacuum dryer connected to a portable air compressor (GAST. INC)] set at relative vacuum degree of −25 kPa for tomato. Vacuum pressure was maintained for approximately 10 s and then released rapidly to atmospheric pressure. This operation was repeated once or twice. Treated sprouts were sown in nutritional soil in pots.

After the cotyledon fully developed, the mixture of the Agrobacterium strain containing TRV1 and TRV2 or its derivatives was infiltrated to the cotyledon with 1 mL needle-less syringe to keep the silence effect persist long enough. The plants were used for further study after 2 weeks.

PDS-silenced tomato seedlings are shown in Fig. S2.

### Determination of ethylene emission

The measurement of ethylene production was performed according to Xu *et al*.^8^. Tomato seedlings were placed in 100 mL gas-tight glass vessels and incubated at room temperature (25 °C) for 2 h. Then, 1 mL sample of gas was removed and analyzed with a flame ionization gas chromatograph (Agilent 6890 Series GC system, Salem, MA, USA) equipped with an activated alumina stainless steel (SS) column. The carrier gas (helium) flow rate was 0.5 mL s^−1^. The detector and injector were operated at 100 °C, and the oven was at 50 °C.

To determine ACC content and ACS activity, tomato leaves were extracted according to the methods described by Wang *et al*. 1992 with slight modifications^58^. Tissues from tomato seedling leaves were extracted in 1 volume of 1.0 M potassium phosphate buffer (pH 8.0) containing 0.5 mM pyridoxal-5-phosphate and 4.0 mM DTE. ACO activity was measured according to Bulens *et al*.^59^.

### Detection of ROS accumulation

ROS accumulation was also detected using the fluorescent probe 2′,7′-dichlorofluorescin diacetate (H_2_DCFDA) (Sigma-Aldrich). Briefly, leaf disks were first vacuum-infiltrated (twice for 5 min) in 10 μM of H_2_DCFDA and observed under a Nikon Eclipse E600 epifluorescence microscope (Nikon, Tokyo, Japan) equipped with a Nikon B-2A filter block (450–490 nm excitation filter, 505 nm dichroic mirror, 520 nm barrier filter). The pixel intensities of fluorescence images, acquired using the microscope, were determined by using Image, J-software (NIH, USA).

H_2_O_2_ content was detected by the Amplex red hydrogen peroxide/peroxidase assay kit (Invitrogen).

### CO_2_ assimilation and Chlorophyll content measurements

The CO_2_ assimilation rate (net photosynthetic rate, Pn) was determined using an open system (TPS-1, PP system, UK) at a CO_2_ content of 360 cm^3^ m^−3^, 70% relative humidity. The total chlorophyll content of leaf was extracted with 80% acetone from the fresh leaves and measured according to Lichtenthaler & Wellburn^60^.

### RNA extraction and qRT-PCR

Total RNA was extracted from *Solanum lycopersicum* leaves using Trizol reagent (Invitrogen, Shanghai, China) according to the manufacturer’s recommendations, followed by further purification with DNase I treatment before PCR. First-strand cDNA was synthesized using Moloney murine leukemia virus reverse transcriptase (Invitrogen). Quantitative real-time PCR analysis was performed on an iCycler (Bio-Rad, Beijing, China) using the comparative *C*_t_ (threshold cycle) method^9^. *ACTIN* was used as the internal control. All of the qRT-PCR primers are listed in Supplementary Table S1. At least three biological replicates were performed for each sample and at least three technical replicates were analyzed for each biological replicate.

### Analysis of chlorophyll fluorescence

Chlorophyll fluorescence was determined with an imaging pulse amplitude modulated fluorometer (IMAG-MINI, Heinz Walz, GER). For F_v_/F_m_ measurement, plants were dark adapted for 30 min. Maximal fluorescence (F_m_) was measured by a 0.8 s pulse of light at about approximately 4 μmolm^−2^ s^−1^, minimal fluorescence (F_o_) was measured during the weak measuring pulses. Chlorophyll fluorescence quenching and ΦPS II were measured as previously described, respectively.

### Superoxide and H_2_O_2_ staining

Superoxide and H_2_O_2_ staining were visually detected with nitro blue tetrazolium (NBT) and 3,3′-diaminobenzidine (DAB). Tomato leaves were vacuum infiltrated with NBT (0.5 mg ml^−1^) solutions for 2 h or DAB (2 mg ml^−1^) solutions for 8 h. Leaves were then decolorized in boiling ethanol (95%) for 15 min.

### Oxidative damage estimation

Leaf relative water content (RWC) is defined in the equation: RWC = (F_w_ − D_w_)/(T_w_ − D_w_) × 100%. F_w_ represents fresh leaf weight, D_w_ represents dry leaf weight and T_w_ represents turgid leaf weight. Electrolyte leakage (EL) was measured according to Deng *et al*.^9^. The different samples were boiled at 100 °C for 10 min to achieve 100% electrolyte leakage after measuring electrical conductivity. The relative conductivity of plasma membranes was calculated based on the ratio of electrical conductivity before and after boiling. Lipid peroxidation was estimated by measuring the malondialdehyde (MDA) as previously described^61^. Trypan Blue staining was performed to show cell death.

### Enzyme activity assays

For the enzyme assays, 500 mg of leaf samples were homogenized in 5 ml 25 mM PBS buffer (pH = 7.8) containing 0.2 mM EDTA, 2 mM ASA and 2% PVP. The homogenate was centrifuged at 12 000 g for 20 min at 4 °C, and the supernatant was immediately used for the determination of enzymatic activity. SOD CAT and APX activity, was measured according to Deng *et al*.^57^, by monitoring the decrease in absorbance at 290 nm as ascorbate was oxidized. Glutathione reductase (GR) activity was detected following the modified method (Meloni *et al*.^62^).

### Statistical analysis

Statistical analysis of the results from experiments with three or more mean values was performed using a one-way ANOVA as dictated by the number of main effects. The difference was considered to be statistically significant when P < 0.05.

## Additional Information

**How to cite this article**: Zhu, T. *et al*. Ethylene and hydrogen peroxide are involved in brassinosteroid-induced salt tolerance in tomato. *Sci. Rep.* **6**, 35392; doi: 10.1038/srep35392 (2016).

## Supplementary Material

Supplementary Information

## References

[b1] Kagale S., Divi U. K., Krochko J. E., Keller W. A. & Krishna P. Brassinosteroid confers tolerance in Arabidopsis thaliana and Brassica napus to a range of abiotic stresses. Planta 225, 353–364 (2007). 16906434 10.1007/s00425-006-0361-6

[b2] Lorenzo O. & Solano R. Molecular players regulating the jasmonate signaling network. Curr. Opin. Plant Biol. 8, 532–540 (2005). 16039901 10.1016/j.pbi.2005.07.003

[b3] Licausi F., Takagi M. O. & Perata P. APETALA2/Ethylene Responsive Factor (AP2/ERF) transcription factors: mediators of stress responses and developmental programs. New Phytol. 199, 639–649 (2013). 24010138 10.1111/nph.12291

[b4] Ha S., Vankova R., Yamaguchi-Shinozaki K., Shinozaki K. & Tran L. Cytokinins: metabolism and function in plant adaptation to environmental stresses. Trends Plant Sci. 17, 172–179 (2012). 22236698 10.1016/j.tplants.2011.12.005

[b5] Tognetti V. B., Mühlenbock P. & Van Breusegem F. Stress homeostasis-the redox and auxin perspective. Plant Cell Environ. 35, 321–333 (2012). 21443606 10.1111/j.1365-3040.2011.02324.x

[b6] Rivas-San, Vicente, M. & Plasencia, J. Salicylic acid beyond defence: its role in plant growth and development. J. Exp. Bot. 62, 3321–3338 (2011). 21357767 10.1093/jxb/err031

[b7] Baxter A., Mittler R. & Suzuki N. ROS as key players in plant stress signalling. J. Exp. Bot. 65, 1229–1240 (2014). 24253197 10.1093/jxb/ert375

[b8] Xu F. . A novel role for cyanide in the control of cucumber (Cucumis sativus L.) seedlings response to environmental stress. Plant Cell Environ. 35, 1983–1997 (2012). 22554042 10.1111/j.1365-3040.2012.02531.x

[b9] Deng X. G., Zhu T., Zhang D. W. & Lin H. H. The alternative respiratory pathway is involved in brassinosteroid-induced environmental stress tolerance in *Nicotiana benthamiana*. J. Exp. Bot. 66, 6219–6232 (2015). 26175355 10.1093/jxb/erv328 PMC4588879

[b10] Divi U. K., Rahman T. & Krishna P. Brassinosteroid-mediated stress tolerance in Arabidopsis shows interactions with abscisic acid, ethylene and salicylic acid pathways. BMC Plant Bio. 10, 151 (2010). 20642851 10.1186/1471-2229-10-151 PMC3095295

[b11] Wang Z. Y. Brassinosteroids modulate plant immunity at multiple levels. P. Natl. Acad. Sci. USA. 109, 7–8 (2012). 10.1073/pnas.1118600109 PMC3252925 22198764

[b12] Li J. & Chory J. A putative leucine-rich repeat receptor kinase involved in brassinosteroid signal transduction. Cell 90, 929–938 (1997). 9298904 10.1016/s0092-8674(00)80357-8

[b13] Wang Z. Y. . Nuclear-localized BZR1 mediates brassinosteroid-induced growth and feedback suppression of brassinosteroid biosynthesis. Dev. Cell 2, 505–513 (2002). 11970900 10.1016/s1534-5807(02)00153-3

[b14] Yin Y. H. . BES1 accumulates in the nucleus in response to brassinosteroids to regulate gene expression and promote stem elongation. Cell 109, 181–191 (2002). 12007405 10.1016/s0092-8674(02)00721-3

[b15] Wang W. F., Bai M. Y. & Wang Z. Y. The brassinosteroid signaling network-a paradigm of signal integration. Curr. Opin. Plant. Boil. 21, 147–153 (2014). 10.1016/j.pbi.2014.07.012 PMC4400584 25139830

[b16] Zhu T. . Effects of brassinosteroids on quality attributes and ethylene synthesis in postharvest tomato fruit. Postharvest Boil. Tec. 100, 196–204 (2015).

[b17] Richard N. A. & Jeannette M. A. Effects of brassinosteroid, auxin, and cytokinin on ethylene production in Arabidopsis thaliana plants. J. Exp. Bot. 59, 3019–3026 (2008). 18583350 10.1093/jxb/ern159 PMC2504343

[b18] Li X. J. . DWARF overexpression induces alteration in phytohormone homeostasis, development, architecture and carotenoid accumulation in tomato. Plant Biotechnol. J. 14, 1021–1033 (2016). 26383874 10.1111/pbi.12474 PMC11388817

[b19] Guo H. & Ecker J. R. The ethylene signaling pathway: new insights. Curr. Opin. Plant Biol. 7, 40–49 (2004). 14732440 10.1016/j.pbi.2003.11.011

[b20] Klee H. & Tieman D. The tomato ethylene receptor gene family: form and function. Physiol. Plantarum 115, 336–341 (2002). 10.1034/j.1399-3054.2002.1150302.x 12081525

[b21] Leclercq J. . LeCTR1, a tomato CTR1-like gene, demonstrates ethylene signaling ability in Arabidopsis and novel expression patterns in tomato. Plant Physiol. 130, 1132–1142 (2002). 12427980 10.1104/pp.009415 PMC166634

[b22] Ecker J. R. The ethylene signal transduction pathway in plants. Science 268, 667–675 (1995). 7732375 10.1126/science.7732375

[b23] O’Donnell P. J. . Ethylene as a signal mediating the wound response of tomato plants. Science 274, 1914–1917 (1996). 8943205 10.1126/science.274.5294.1914

[b24] Penninckx I. A. . Pathogen-induced systemic activation of a plant defenses in gene in Arabidopsis follows a salicylic acid-independent pathway. Plant Cell 8, 2309–2323 (1996). 8989885 10.1105/tpc.8.12.2309 PMC161354

[b25] Yi H. C. . Auxin and brassinosteroid differentially regulate the expression of the three members of the 1-aminocyclopropane-1-carboxylate synthase family in mung bean (*Vigna radiata L.*). Plant Mol. Biol. 41, 443–454 (1999). 10608655 10.1023/a:1006372612574

[b26] Hansen M., Chae H. S. & Kieber J. J. Regulation of ACS protein stability by cytokinin and brassinosteroid. Plant J. 57, 606–614 (2009). 18980656 10.1111/j.1365-313X.2008.03711.x PMC2807401

[b27] Symons G. M. . Grapes on steroids. Brassinosteroids are involved in grape berry ripening. Plant Physiol. 140, 150–158 (2006). 16361521 10.1104/pp.105.070706 PMC1326039

[b28] Liu L. . Ectopic expression of a BZR1-1D transcription factor in brassinosteroid signalling enhances carotenoid accumulation and fruit quality attributes in tomato. Plant biotechnol. J. 12, 105–115 (2014). 24102834 10.1111/pbi.12121

[b29] De Grauwe L., Vandenbussche F., Tietz O., Palme K. & Van Der Straeten D. Auxin, ethylene and brassinosteroids: tripartite control of growth in the Arabidopsis hypocotyl. Plant Cell Physiol. 46, 827–836 (2005). 15851402 10.1093/pcp/pci111

[b30] Deslauriers S. D. & Larsen P. B. FERONIA is a key modulator of brassinosteroid and ethylene responsiveness in Arabidopsis hypocotyls. Mol. Plant 3, 626–640 (2010). 20400488 10.1093/mp/ssq015

[b31] Cai Z. . GSK3-like kinases positively modulate abscisic acid signaling through phosphorylating subgroup III SnRK2s in Arabidopsis. P. Natl. Acad. Sci. USA 111, 9651–9656 (2014). 10.1073/pnas.1316717111 PMC4084465 24928519

[b32] Zimmermann P., Hirsch-Hoffmann M., Hennig L. & Gruissem W. GENEVESTIGATOR. Arabidopsis microarray database and analysis toolbox. Plant Physiol. 136, 2621–2632 (2004). 15375207 10.1104/pp.104.046367 PMC523327

[b33] Kazan K. Diverse roles of jasmonates and ethylene in abiotic stress tolerance. Trends Plant Sci. 20, 1360–1385 (2015). 10.1016/j.tplants.2015.02.001 25731753

[b34] Xia X. J. . Induction of systemic stress tolerance by brassinosteroid in *Cucumis sativus*. New Phytol. 191, 706–720 (2011). 21564100 10.1111/j.1469-8137.2011.03745.x

[b35] Zhou J. . H_2_O_2_ mediates the crosstalk of brassinosteroid and abscisic acid in tomato responses to heat and oxidative stresses. J. Exp. Bot. 65, 4371–4383 (2014). 24899077 10.1093/jxb/eru217 PMC4112640

[b36] Nie W. F. . Silencing of tomato RBOH1 and MPK2 abolishes brassinosteroid-induced H_2_O_2_ generation and stress tolerance. Plant Cell Environ. 36, 789–803 (2013). 22994632 10.1111/pce.12014

[b37] Cano-Delgado A. . BRL1 and BRL3 are novel brassinosteroid receptors that function in vascular differentiation in Arabidopsis. Development 131, 5341–5351 (2004). 15486337 10.1242/dev.01403

[b38] Youssef B. & Yvon J. The molecular circuitry of brassinosteroid signaling. New Phytol. 206, 522–540 (2014). 10.1111/nph.13269 25615890

[b39] Li J. . BAK1, an Arabidopsis LRR receptor-like protein kinase, interacts with BRI1 and modulates brassinosteroid signaling. Cell 110, 213–222 (2002). 12150929 10.1016/s0092-8674(02)00812-7

[b40] Wu W. . Somatic embryogenesis receptor-like kinase 5 in the ecotype Landsberg erecta of Arabidopsis is a functional RD LRR-RLK in regulating brassinosteroid signaling and cell death control. Front. Plant Sci. 6, 852 (2015). 26528315 10.3389/fpls.2015.00852 PMC4606071

[b41] Ge X. M. . Heterotrimeric G protein mediates ethylene-induced stomatal closure via hydrogen peroxide synthesis in Arabidopsis. Plant J. 82, 138–150 (2015). 25704455 10.1111/tpj.12799

[b42] Vandenabeele S. . A comprehensive analysis of hydrogen peroxide-induced gene expression in tobacco. P. Natl. Acad. Sci. USA 100, 16113–16118 (2003). 10.1073/pnas.2136610100 PMC307701 14671332

[b43] Deng X. G. . Role of brassinosteroid signaling in modulating Tobacco mosaic virus resistance in Nicotiana benthamiana. Sci. Rep. 6, 20579 (2016). 26838475 10.1038/srep20579 PMC4738339

[b44] Deng X. G. . Orchestration of hydrogen peroxide and nitric oxide in brassinosteroids mediated systemic virus resistance in Nicotiana benthamiana. Plant J. 85, 478–493 (2016). 26749255 10.1111/tpj.13120

[b45] Neill S., Desikan R. & Hancock J. Hydrogen peroxide signalling. Curr. Opin. Plant Biol. 5, 388–395 (2002). 12183176 10.1016/s1369-5266(02)00282-0

[b46] Jaspers P. & Kangasjärvi J. Reactive oxygen species in abiotic stress signaling. Physiol. Plantarum 138, 405–413 (2010). 10.1111/j.1399-3054.2009.01321.x 20028478

[b47] Bajguz A. & Hayat S. Effects of brassinosteroids on the plant responses to environmental stresses. Plant Physiol. Bioch. 47, 1–8 (2009). 10.1016/j.plaphy.2008.10.002 19010688

[b48] Alonso J. M., Hirayama T., Roman G., Nourizadeh S. & Ecker J. R. EIN2, a bifunctional transducer of ethylene and stress responses in Arabidopsis. Science 284, 2148–2152 (1999). 10381874 10.1126/science.284.5423.2148

[b49] Guo H. & Ecker J. R. Plant responses to ethylene gas are mediated by SCF(EBF1/EBF2)-dependent proteolysis of EIN3 transcription factor. Cell 115, 667–677 (2003). 14675532 10.1016/s0092-8674(03)00969-3

[b50] Peng J. Y. . Salt-Induced Stabilization of EIN3/EIL1 Confers Salinity Tolerance by Deterring ROS Accumulation in Arabidopsis. Plos Genet. 10, 10 (2014). 10.1371/journal.pgen.1004664 PMC4199496 25330213

[b51] Tieman D. M., Ciardi J. A., Taylor M. G. & Klee H. J. Members of the tomato *LeEIL* (EIN3-like) gene family are functionally redundant and regulate ethylene responses throughout plant development. Plant J. 26, 47–58 (2001). 11359609 10.1046/j.1365-313x.2001.01006.x

[b52] Yokotani N., Tamura S., Nakano R., Inaba A. & Kubo Y. Characterization of a novel tomato EIN3-like gene (*LeEIL4*). J. Exp. Bot. 54, 2775–2776 (2003). 14623944 10.1093/jxb/erg308

[b53] Kim T. W., Michniewicz M., Bergmann D. C. & Wang Z. Y. Brassinosteroid regulates stomatal development by GSK3-mediated inhibition of a MAPK pathway. Nature 482, 419–422 (2012). 22307275 10.1038/nature10794 PMC3292258

[b54] Khan M. . Brassinosteroid-regulated GSK3/Shaggy-like kinases phosphorylate mitogen-activated protein (MAP) kinase kinases, which control stomata development in Arabidopsis thaliana. J. Biol. Chem. 288, 7519–7527 (2013). 23341468 10.1074/jbc.M112.384453 PMC3597792

[b55] Yoo S. D., Cho Y. H., Tena G., Xiong Y. & Sheen J. Dual control of nuclear EIN3 by bifuracate MAPK cascades in C_2_H_2_ signalling. Nature, 451, 789–795 (2008). 18273012 10.1038/nature06543 PMC3488589

[b56] Zhao Q. & Guo H. W. Paradigms and paradox in the ethylene signaling pathway and interaction network. Mol. Plant 4, 626–634 (2011). 21690206 10.1093/mp/ssr042

[b57] Yan H. X. . Sprout vacuum-infiltration: a simple and efficient agroinoculation method for virus-induced gene silencing in diverse solanaceous species. Plant Cell Rep. 31, 1713–1722 (2012). 22717672 10.1007/s00299-012-1285-1

[b58] Wang T. W. & Arteca R. N. Effects of low O_2_ root stress on ethylene biosynthesis in tomato plants (*Lycopersicon esculentum* Mill cv Heinz 1350). Plant Physiol. 98, 97–100 (1992). 16668654 10.1104/pp.98.1.97 PMC1080154

[b59] Bulens I. . Protocol: an updated integrated methodology for analysis of metabolites and enzyme activities of ethylene biosynthesis. Plant Methods 7, 1–10 (2011). 21696643 10.1186/1746-4811-7-17 PMC3142538

[b60] Lichtenthaler H. K. & Wellburn A. R. Determinations of total carotenoids and chlorophylls a and b of leaf extracts in different solvents. Biochem. Soc. T. 11, 591–592 (1983).

[b61] Zhu F. . Salicylic acid and jasmonic acid are essential for systemic resistance against tobacco mosaic virus in *Nicotiana benthamiana*. Mol. Plant Microbe. In. 27, 567–577 (2014). 10.1094/MPMI-11-13-0349-R 24450774

[b62] Meloni D. A., Oliva M. A., Martinez C. A. & Cambraia J. Photosynthesis and activity of superoxide dismutase, peroxidase and glutathione reductase in cotton under salt stress. Environ. Exp. Bot. 49, 69–76 (2003).

