# Supplementary material for: Ethylene and hydrogen peroxide are involved in brassinosteroid-induced salt tolerance in tomato
Source: Sci Rep. 2016 Oct 14;6:35392. doi: 10.1038/srep35392 (PMC5064326; doi:10.1038/srep35392)
Supplement: Supplementary Information [file srep35392-s1.pdf]

SUPPLEMENTARY INFORMATION

Ethylene and hydrogen peroxide are involved in brassinosteroid-induced salt tolerance in tomato

Tong Zhu[1], Xing-Guang Deng[1], Xue Zhou[1], Li-Sha Zhu, Li-juan Zou, Peng-Xu Li, Da-Wei Zhang* & Hong-Hui Lin*

Supplementary Figures

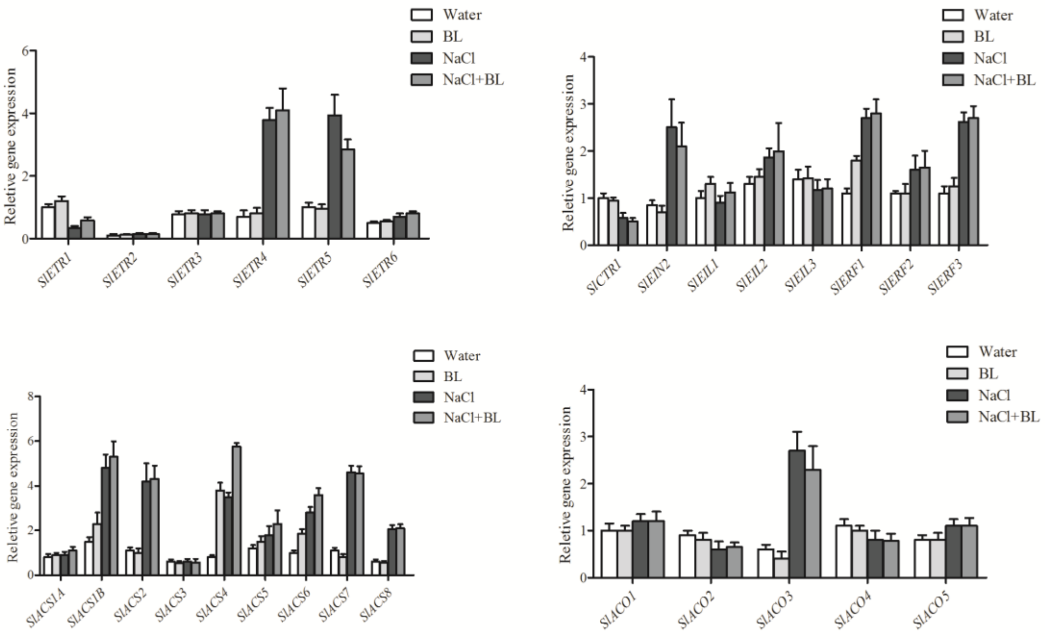

Supplementary materials Figure. S1 The transcription level of ethylene synthesis and ethylene signaling pathway genes. Bars represent mean and standard deviation of values obtained from three biological repeats.

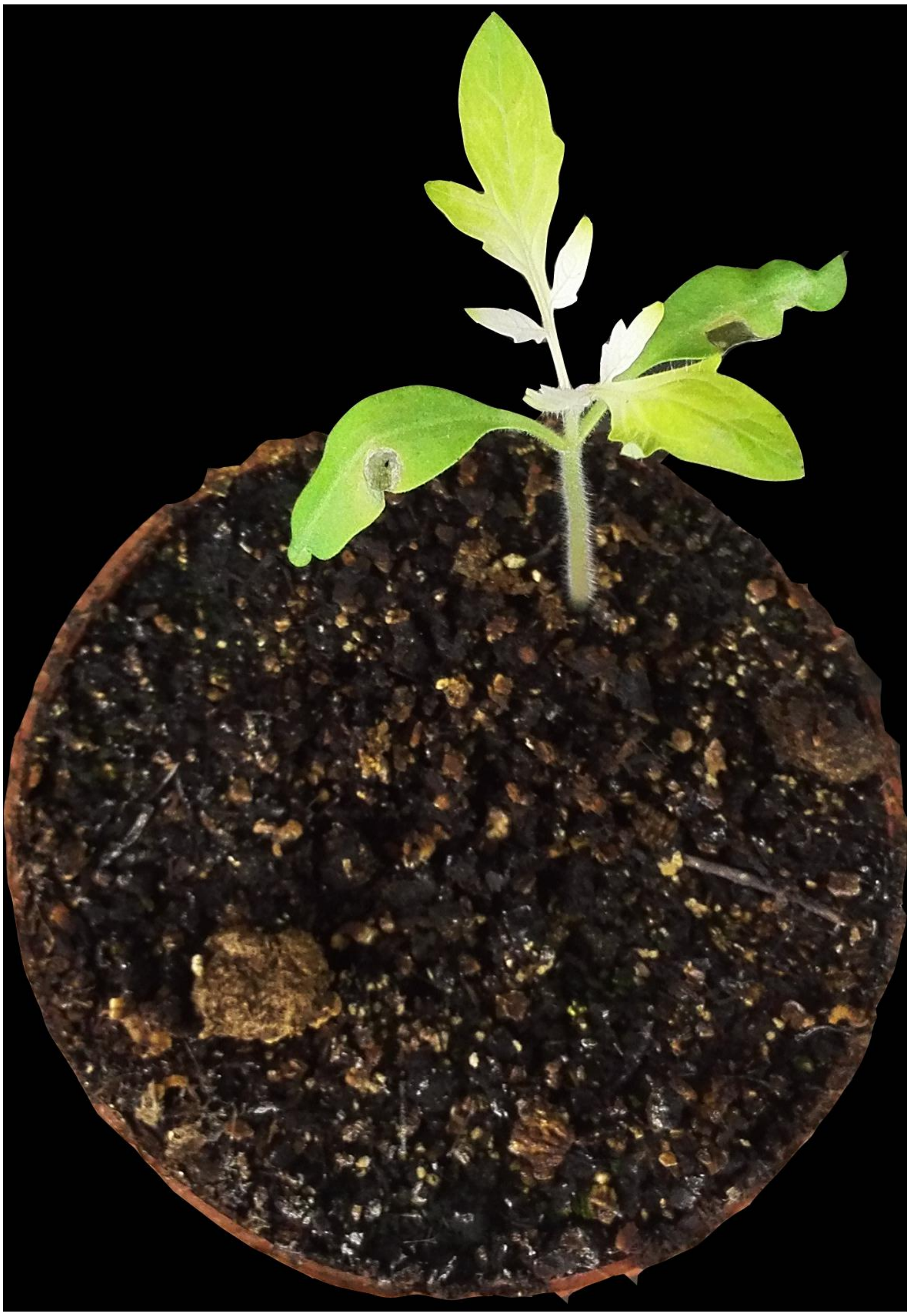

Supplementary materials Fig. S2 TRV:*SlPDS* tomato seedlings. 2 weeks after cotyledon infiltration.

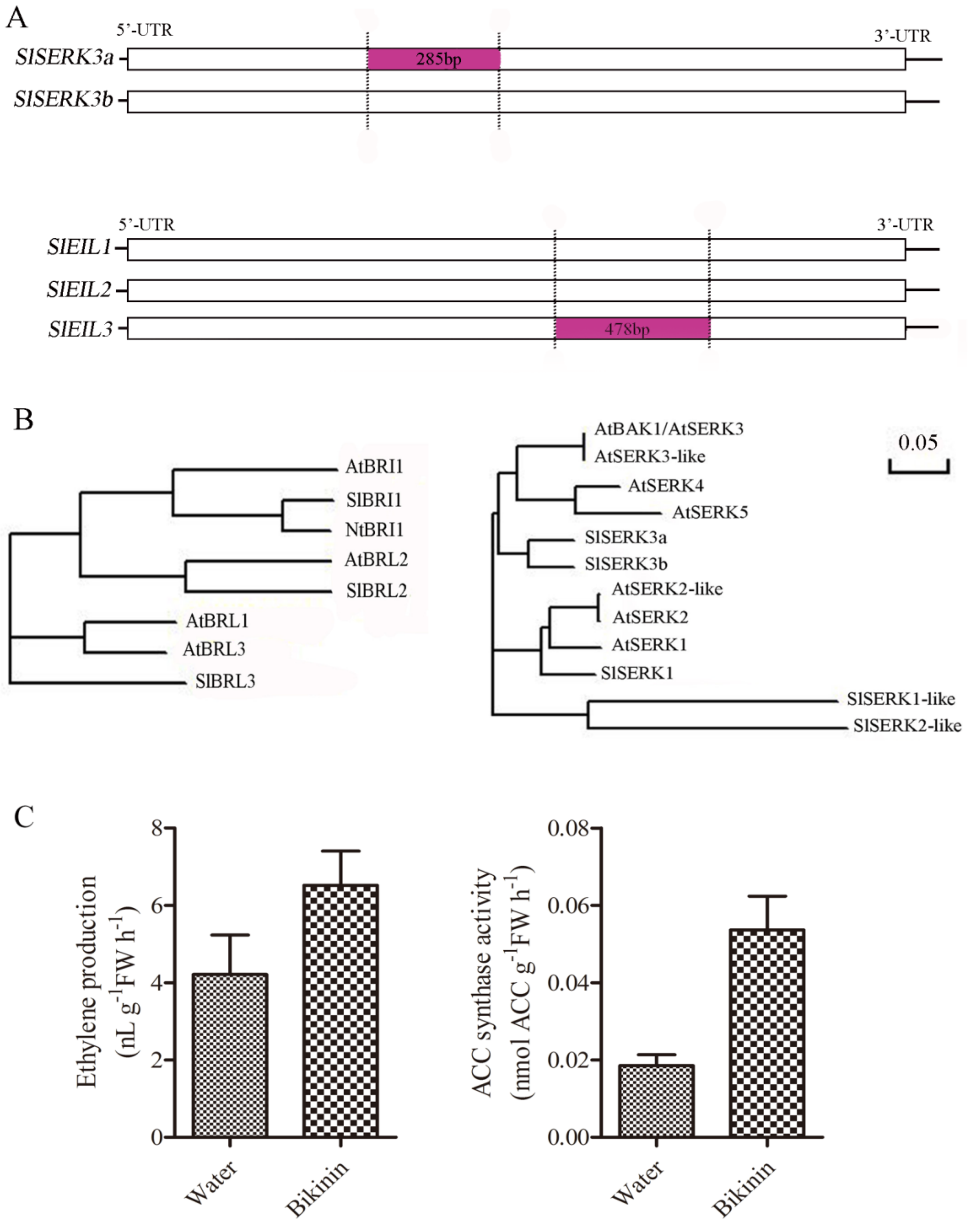

Supplementary materials Fig. S3 Locations of the VIGS inducing fragments in tomato *SlBAK1*/*SlSERK3* and *SlEIL* gene families. The vertical dotted lines showed the putative target gene(s) of these silencing constructs. The purple boxes indicate the location and length of the silencing fragment (A). Phylogenetic analysis showed the BRI1 and BAK1 and their homologs in tomato, including several BRL and SERK-like genes (B).Effects of 10μM bikinin on ethylene emission and ACS activity (C) .

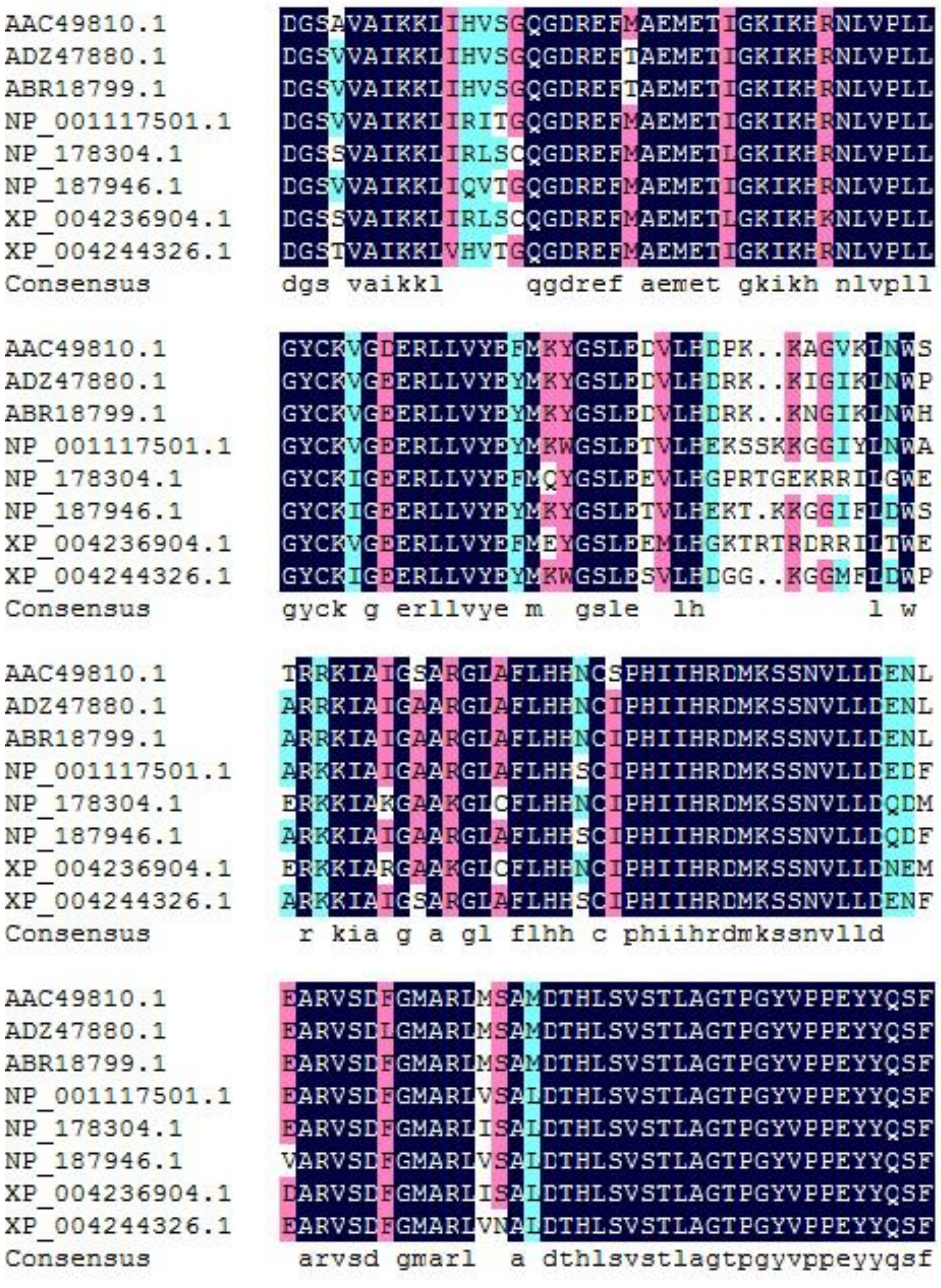

Supplementary materials Fig. S4 Conservative degree analysis of tomato BRI1 family and its homologs domain alignment.

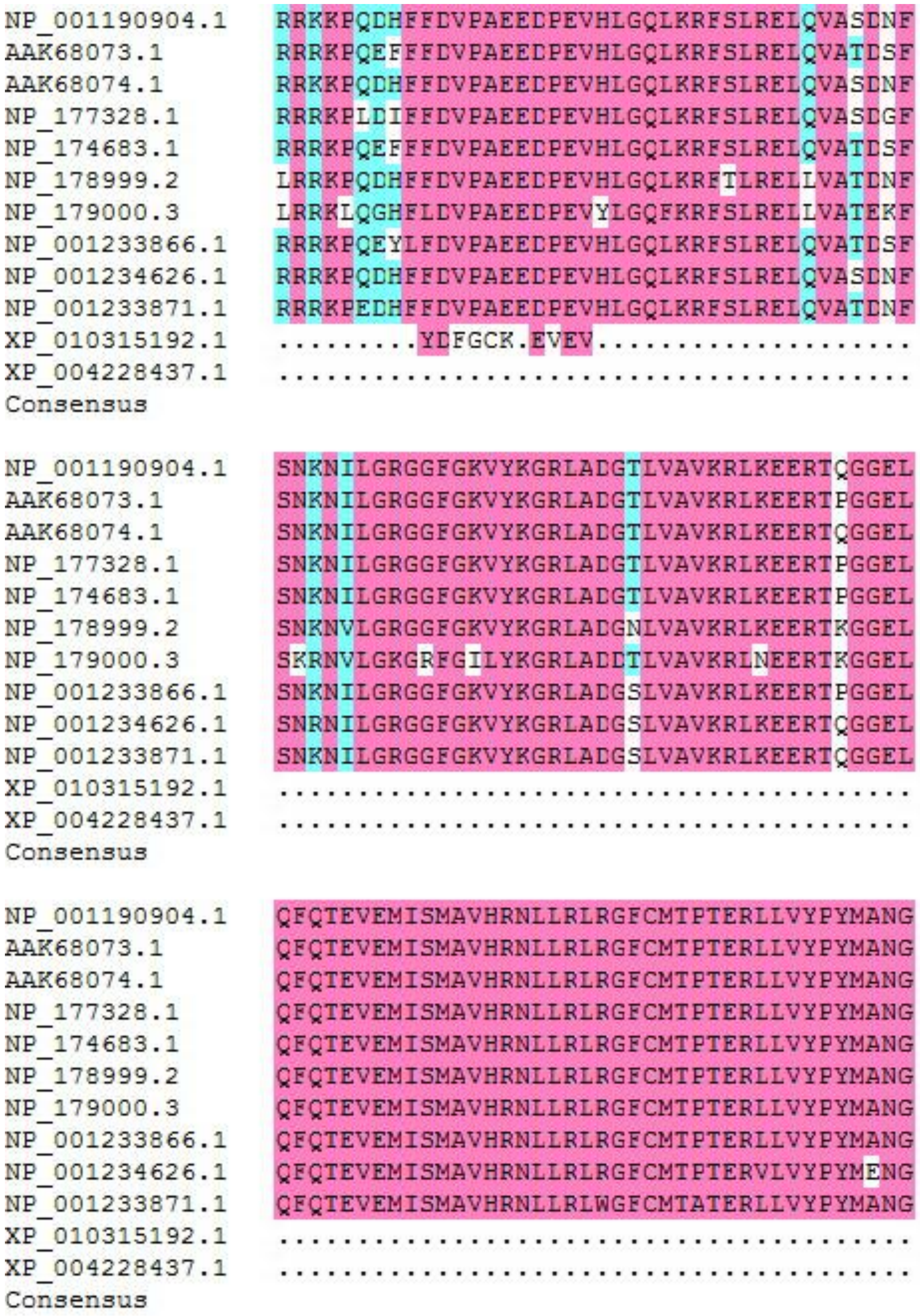

Supplementary materials Fig. S5 Conservative degree analysis of tomato SERK family and its homologs domain alignment.

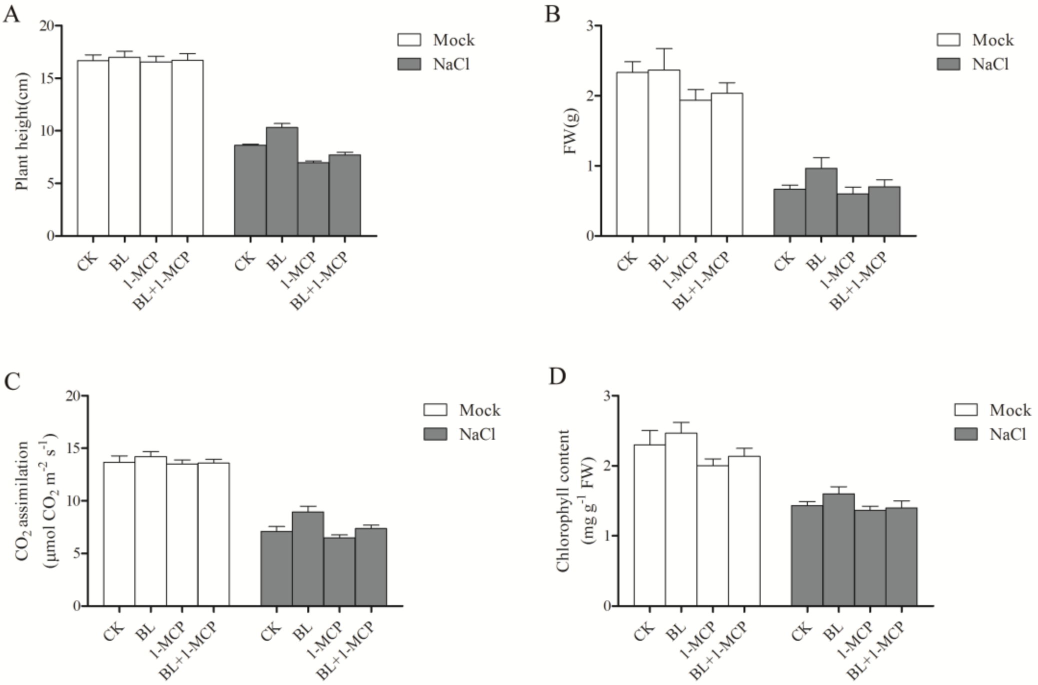

Supplementary materials Fig. S6 The growth condition after 1-MCP treatment of tomato seedlings. Plant height (A), fresh weight (FW) (B), CO_2 assimilation rate (C) and chlorophyll content (D) of the silenced plants determined after 21 d of water or NaCl treatment with or without 0.1μM BL pretreatment. Bars represent the mean and standard deviation of values obtained from three biological repeats. Significant differences ($P<0.05$) are denoted by different lowercase letters. FW, fresh weight.

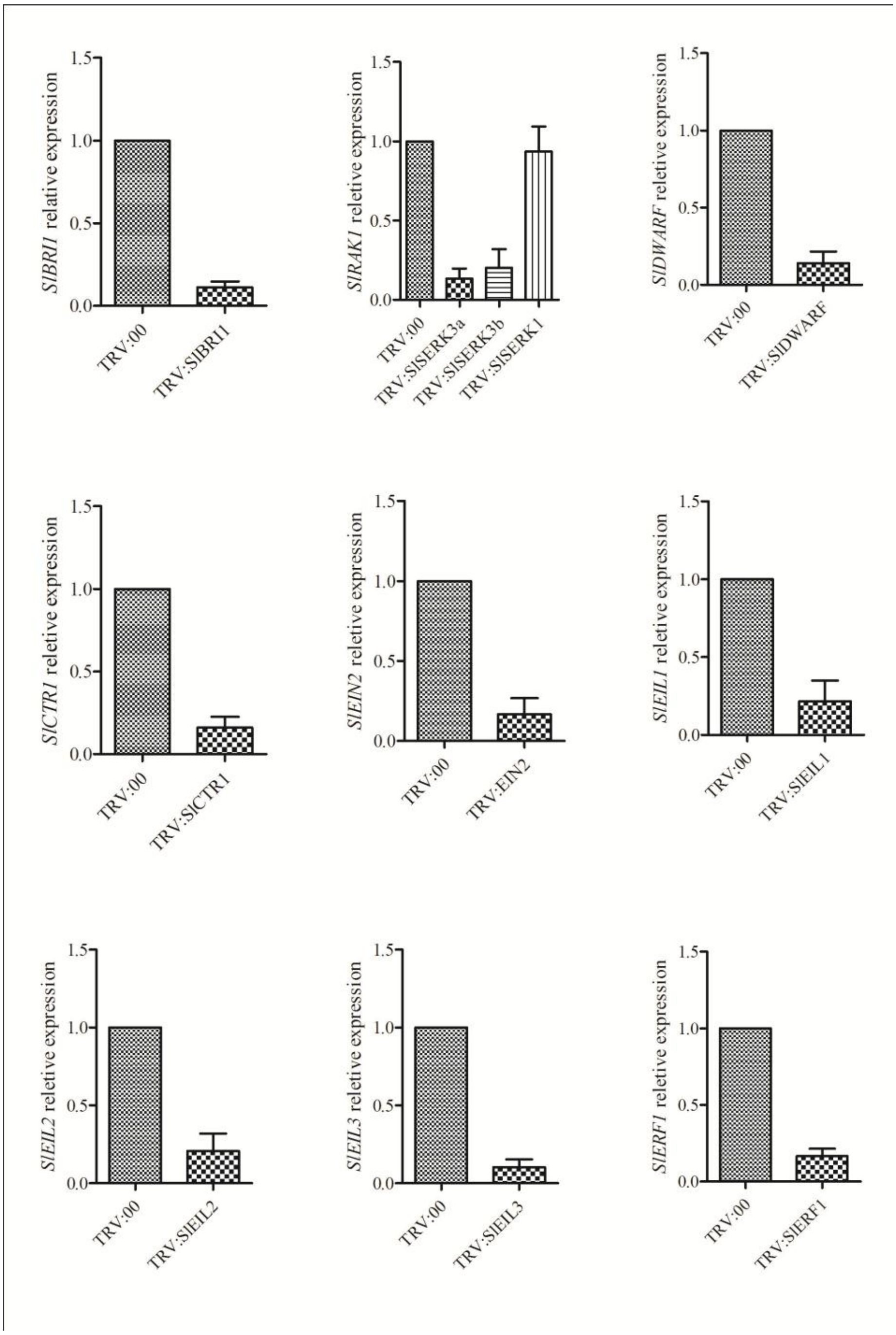

Supplementary materials Fig. S7 Confirmation of the *SlBRI1*, *SlBAK1*, *SlDWARF*, *SlCTR1*, *SlEIN2*, *SlEIL1-3* and *SlERF1* genes silencing in tomato plants. Real-time PCR analysis was conducted using total RNA extracted from the sixth leaves of tomato plants inoculated with Agrobacterium GV3101 carrying TRV-target genes and the corresponding non-silenced leaves of TRV:00 infected control plants. Bars represent mean and standard deviation of values obtained from three biological repeats.

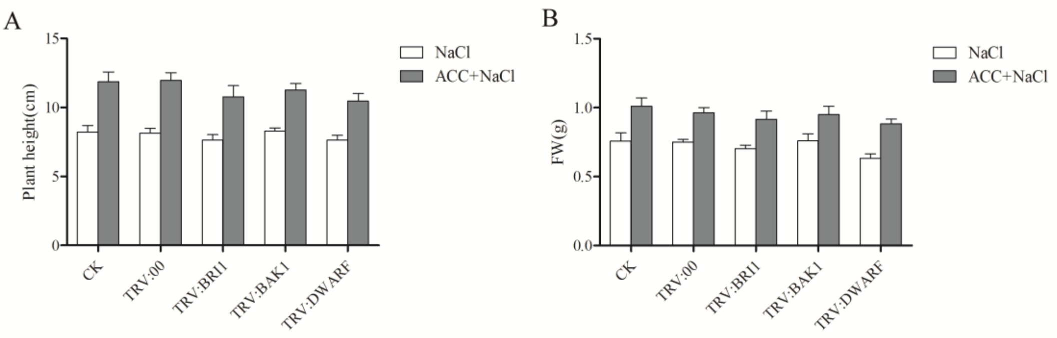

Supplementary materials Fig. S8 The growth condition of *SlBRI1*-, *SlBAK1* and *SlDWARF*-silenced treated with water or 200 mM NaCl for 21 d. The seedlings were treated with water or 200μM ACC from the cotyledon stage to the six-leaf stage under salt condition. Plant height (A), fresh weight (FW) (B) of the silenced plants determined after 21 d of water or NaCl treatment with or without ACC pretreatment. Bars represent the mean and standard deviation of values obtained from three biological repeats. Significant differences ($P<0.05$) are denoted by different lowercase letters. FW, fresh weight.

Primers for VIGS assay:

Gene	Accession number	Forward primer (5'-3')	Reverse primer (5'-3')
SlBRI1	HQ699335	TCCCTCACCAGTCTTACAT	CTTCCTCCTCCTCTTCTTC
SlBAK1	NM_001247697	TCTTCCTCCTACACCCTC	ACCTTACCAAATCCACCT
SlDWARF	NM_001247334	ACTGTTTCCACCACTTCT	GTGTTCCAGGCTCTTATC
SlCTR1	XM_004238493	ACCAAACGCCCTCATCTA	GCTACAGCACCAACTACC
SlEIN2	AY566238	CCTGCTGTCGTTCATCCT	CTTCGCTTCTGCTGTTGC
SlEILs	AF328786	GTGGAGGGATCGAATG	TACCGCCGTCAGAACA
SlERF1	NM_001247912	AAAGATGTCAAGCCCACT	GTTCCTAACCAAACCCTA

Primers for qRT-PCR

Gene	Accession number	Forward primer (5'-3')	Reverse primer (5'-3')
SlETR1	AF043084	TAGCAACGCTCATCAACGA	AGCCCAGTGCCACCAGAAT
SlETR2	AF043085	TAGCAGGATGGTAACGAAG	ACAGTCCCGTGCTCTAAAA
SlETR3	NM_001246965	TTCTGGTTCTCCCGATGA	GAAGATTGAATGTTCCGTTT
SlETR4	AF118843	GATGGATGAAGGCTTGAGT	ACAGCAGGGCTAAGAACAC
SlETR5	NM_001247283	GGTGGCTGTGGCTTTATCC	AGGGCTTCATTTCTACTGG

SlETR6	NM_001247221	CTCCTCCAACATACGACAC	CTTCAAAGCCATCTAAATCA
SlCTR1	XM_004238493	ATGGAGCCTGGAGGTAGAA	TGATAGATGAGGGCGTTTG
SlEIN2	AY566238	TATGCGGATTCCAGCGACTT	CTTGGCGAGGAACATAGCG
SlEIL1	AF328784	TGAAGATGGGCAAAGGATG	TGGAAGTTGTCGTTGGAGC
SlEIL2	AF328785	GCTCCACCCTCCTTTGATA	GAGGCATCTTGCTTCGTCA
SlEIL3	NM_001247617	GCCTGGAAGGTTGGTGTT	GCTGTATGGGCAGTGAAG
SlERF1	NM_001247919	GTCAACTTGGGAGTGGGAGTA	ACAAATCGGGAACGGCTAT
SlERF2	NM_001247379	CCTCCCTTGAACATTGCTT	TGATTGCCCGTCAACATAC
SlERF3	AY559314	GATTCATCATCGCCGTTAG	TCGTCTTCATCGTCGTCTC
SlACS1A	NM_001246993	ATAGTAATGAGTGGAGGAG	TACAATGTCACGGAGTG
SlACS1B	U72390	GTAATGAGTGGAGGAGCA	CAATGTCTCGTAGCGTGT
SlACS2	NM_001247249	TTATCCAAATGGGTCT	CTAACTCTTCCTCCTCT
SlACS3	NM_001247097	GACCAACCCTTCAAATC	GCAGTTAATACCAGCAC
SlACS4	NM_001247351	TCGGAGGTAGGATGGTTT	TACGAGCGAGGAATTGGA
SlACS5	NM_001247227	TAACGCTGGATTGTTCTGT	TCCTTCTCCTCCTTGACCC
SlACS6	NM_001247235	AAGCCTTAGAAGAAGCATA	TGTACGAGTAAATAATCCC
SlACS7	NM_001247417	TCCCACTCCATACTACCCT	GAACTCTATCCCATACATCA
SlACS8	NM_001247231	CGGGATTTGATCGAGACT	ACTAACGAACTTGGGTGA
SlACO1	NM_001247095	ATGTCCTAAGCCCGATTT	GTGTCCCGTCTGTTTGTG
SlACO2	Y00478	AACMTCGCTTGATWACAA	ATGGGKYWTKGAAYAACT
SlACO3	NM_001309213	CTTTGAGCAAGATAACGAGGT	GCTGAGGACATGGTGGGT
SlACO4	NM_001246938	ACTATCCTCCTTGTCCCA	AGTCTCCACAGCCTTCAT
SlACO5	NM_001247108	TTTTCAGGCAGCAAGGGTC	AAGCGGAGGTGACAGGGA

Supplementary Table S1 Primers used for construction of VIGS vectors and real-time PCR analysis
